# Comparing Ligninolytic Capabilities of Bacterial and Fungal Dye-Decolorizing Peroxidases and Class-II Peroxidase-Catalases

**DOI:** 10.3390/ijms22052629

**Published:** 2021-03-05

**Authors:** Dolores Linde, Iván Ayuso-Fernández, Marcos Laloux, José E. Aguiar-Cervera, Antonio L. de Lacey, Francisco J. Ruiz-Dueñas, Angel T. Martínez

**Affiliations:** 1 Centro de Investigaciones Biológicas “Margarita Salas” (CIB), CSIC, Ramiro de Maeztu 9, E-28040 Madrid, Spain; lolalinde@cib.csic.es (D.L.); ivan.ayuso-fernandez@nmbu.no (I.A.-F.); marcos.laloux@gmail.com (M.L.); joseac93@hotmail.com (J.E.A.-C.); fjruiz@cib.csic.es (F.J.R.-D.); 2 Instituto de Catálisis y Petroleoquímica (ICP), CSIC, Marie Curie 2, E-28049 Madrid, Spain; alopez@icp.csic.es

**Keywords:** lignin transformation, dye-decolorizing peroxidases, lignin peroxidases, versatile peroxidases, lignin model dimers, reduction potential, kinetic constants, long-range electron transfer

## Abstract

We aim to clarify the ligninolytic capabilities of dye-decolorizing peroxidases (DyPs) from bacteria and fungi, compared to fungal lignin peroxidase (LiP) and versatile peroxidase (VP). With this purpose, DyPs from *Amycolatopsis* sp., *Thermomonospora curvata*, and *Auricularia auricula-judae*, VP from *Pleurotus eryngii*, and LiP from *Phanerochaete chrysosporium* were produced, and their kinetic constants and reduction potentials determined. Sharp differences were found in the oxidation of nonphenolic simple (veratryl alcohol, VA) and dimeric (veratrylglycerol-β- guaiacyl ether, VGE) lignin model compounds, with LiP showing the highest catalytic efficiencies (around 15 and 200 s^−1^·mM^−1^ for VGE and VA, respectively), while the efficiency of the *A. auricula-judae* DyP was 1–3 orders of magnitude lower, and no activity was detected with the bacterial DyPs. VP and LiP also showed the highest reduction potential (1.28–1.33 V) in the rate-limiting step of the catalytic cycle (i.e., compound-II reduction to resting enzyme), estimated by stopped-flow measurements at the equilibrium, while the *T. curvata* DyP showed the lowest value (1.23 V). We conclude that, when using realistic enzyme doses, only fungal LiP and VP, and in much lower extent fungal DyP, oxidize nonphenolic aromatics and, therefore, have the capability to act on the main moiety of the native lignin macromolecule.

## 1. Introduction

Lignin is a complex polymer formed in the cell wall of vascular plants by radical coupling of three main and several additional aromatic precursors [1]. In the industry, technical lignins obtained as side products of paper pulp mills and bioethanol factories are underused, aside from their burning to obtain energy and recover chemicals [2]. However, with a total production of 100 million tons per year and an estimated annual growth around 2.2% [3], technical lignins can be used as renewable feedstocks to obtain added-value chemical products [4]. In this way, the production of aromatic compounds from lignin exploitation could pump the use of biofuels as an economically sustainable alternative to fossil fuels, and broad the products portfolio of the cellulose industry in agreement with the bio-refinery concept [5]. With this purpose, it is necessary to break down the recalcitrant lignin polymer, which has naturally evolved to confer plants resistance towards physical and microbial agents [6]. For these reasons, studying the specialized organisms being able to cause lignin decay in nature is one of the clues in obtaining efficient biocatalysts for lignin transformation in plant biorefineries [7].

To demonstrate microbial and enzymatic ligninolysis, classical studies used both nonphenolic in vitro synthesized lignin (methylated dehydrogenation polymer, DHP, representing the main nonphenolic moiety in native lignin) and nonphenolic model dimers with the main interunit linkages in lignin (such as β-*O*-4’ alkyl-aryl ether bonds). The most studied lignin degradation systems are those present in white-rot fungi, which secrete a repertoire of oxidoreductase families [7], including: (i) Ligninolytic enzymes in class-II of the peroxidase-catalase superfamily [8], such as lignin peroxidase (LiP), versatile peroxidase (VP) and manganese peroxidase (MnP); (ii) oxidases from the glucose-methanol-choline oxidase/dehydrogenase (GMC) [9] and copper-radical oxidase (CRO) [10,11] (super)families; and (iii) laccases of the multi-copper oxidase (MCO) superfamily [12].

Additionally, degradation of lignin by actinobacteria, α-proteobacteria and γ-proteobacteria has been claimed [13,14,15,16,17]. In this context, dye-decolorizing peroxidases (DyPs), laccases, and glutathione-dependent β-esterases have been reported as involved in bacterial degradation of lignin [18,19,20]. Moreover, in the search of enzymes applicable to industrial processes, better thermostability and more alkaline optimal pH have been reported for bacterial enzymes acting on lignin products than for their fungal counterparts [21,22]. Among them, oxidation of phenolic lignin model dimers by DyPs has been reported, but the activity levels of these enzymes on more recalcitrant nonphenolic models need to be comparatively reassessed [23,24,25]. In fact, only one example has been reported in which extensive conversion of a nonphenolic dimer (veratrylglycerol-β-guaiacyl ether, VGE) is shown, but it was obtained using an extremely high (1:5) enzyme/substrate molar ratio [26] that does not correspond to true enzymatic catalysis. However, being DyPs able to modify phenolic technical lignins, places them as an interesting enzyme family for biotechnological application in the integral use of lignin products in biorefineries.

DyPs are widely distributed in the genomes of fungi, bacteria and archaea [8,27,28,29]. Based on sequence alignments, DyPs are classified into 4 types at the PeroxiBase (now RedoxiBase) database (http://peroxibase.toulouse.inra.fr, accessed on 4 March 2021) with types A, B and C mainly belonging to bacteria and archaea, and type D being of fungal origin. However, a more recent classification based on structure-based alignments [30] divides DyPs into three classes: P (primitive) means former class B, with the most compact structure; I (intermediate) means former class A, with additional sequences; and V (advanced) means former classes C and D, also with additional sequences. Although DyPs are not structurally and evolutionarily related to the peroxidase-catalase superfamily, both display similarities in the heme cavity residues, as well as in the existence of intramolecular long-range electron transfer (LRET) routes for the oxidation of bulky substrates at solvent-exposed oxidation sites [31,32]. However, DyPs are characterized by their high efficiency oxidizing recalcitrant anthraquinonic (e.g., Reactive Blue 5) dyes, with fungal DyPs being often more efficient [33]. From a structural point of view, DyPs share a two-domain ferredoxin-like folding with chlorite dismutases justifying the proposal of a CDE (chlorite dismutase, DyPs and EfeB) structural superfamily [34]. However, Yoshida et al. [30] consider that the evolutionary relationship between the three families is not so clear due to low identity in structure-based alignments, which would make difficult to say whether they represent convergent or divergent evolutionary lines [35]. On other hand, their eco-physiological role also remains uncertain, and the contribution of bacterial DyPs to ligninolysis is controversial, since no agreement on their ability to act on the major nonphenolic moiety of lignin, as shown using model compounds, has been attained.

In this work, two bacterial DyPs from the actinobacteria *Amycolatopsis* sp. (*Asp*DyP2; syn. *Streptomyces setonii*) [36] and *Thermomonospora curvata* (*Tcu*DyP) [37], one fungal DyP from the basidiomycete *Auricularia auricula-judae* (*Aau*DyP) [24,38], and two well-known class-II fungal peroxidases, namely VP from *Pleurotus eryngii* (*Per*VPL) and LiP from *Phanerochaete chrysosporium* (*Pch*LiPA, corresponding to isoenzyme H8) [39], have been comparatively studied. Oxidation of phenolic (guaiacylglycerol-β-guaiacyl ether, GGE) and nonphenolic (VGE) lignin model dimers (Supplementary Figure S1), reduction potential (*E^o^′*) measurements of peroxidase catalytic couples, and structural insights allowed further comparison of these bacterial and fungal peroxidases in the context of lignin biodegradation.

## 2. Results and Discussion

### 2.1. Peroxidase Production

Bacterial *Asp*DyP2 and *Tcu*DyP, and fungal *Aau*DyP, *Per*VPL and *Pch*LiPA were produced in *Escherichia coli* and their kinetic constants on simple and dimeric lignin models (and other substrates), and the *E^o^′* values of redox couples in their catalytic cycles were estimated under standard and comparable conditions. The fungal enzymes were produced as inclusion bodies that were in vitro activated, as described elsewhere [24,40,41], purified to electrophoretic homogeneity, and their molar extinction coefficients used for subsequent enzyme quantification. In contrast, *Asp*DyP2 and *Tcu*DyP were expressed as soluble and active His-tagged proteins, by gene cloning in the pET28 vector. After cell harvest and lysis, these DyPs were purified by affinity chromatography on a Ni-bonded resin, followed by molecular-exclusion and anionic-exchange chromatographic steps for *Asp*DyP2, and *Tcu*DyP, respectively (summaries are included in Supplementary Tables S1 and S2). The second chromatographic step in *Asp*DyP2 isolation does not improve the purification factor (Supplementary Figure S2A) and was omitted in following purifications, but it was important to obtain pure *Tcu*DyP (Supplementary Figure S2B). The identity of purified recombinant *Asp*DyP2 and *Tcu*DyP was confirmed by MALDI TOF/TOF peptide mass fingerprinting after trypsin hydrolysis. The two enzymes show a typical heme-peroxidase spectrum with the Soret band at 405 nm (Supplementary Figure S2C,D) and a Reinheitzahl value (A_405_/A_280_) of 1.5 and 1.2, respectively. Expression of *Tcu*DyP [37], *Asp*DyP2 [36] and a few other type-I [42,43] and type-V [27] bacterial DyPs in *E. coli* with similar spectra and Reinheitzahl values had already been reported previously. 

### 2.2. Kinetics for Oxidation of Simple Peroxidase Substrates 

Firstly, the optimal pH for oxidation of several peroxidase substrates by the heterologously expressed *Asp*DyP2 and *Tcu*DyP was established using low redox-potential dye ABTS, anthraquinonic dye RB19, phenolic compound DMP, nonphenolic compound VA, and divalent Mn^2+^ ion. In the pH 2–9 range, no veratraldehyde was detected from VA with any of the two bacterial DyPs, and Mn^2+^ oxidation was produced with *Asp*DyP2 and not with *Tcu*DyP. A range of pH 3.0–5.0 was optimal for *Asp*DyP2 activity, while *Tcu*DyP displayed a more acidic optimal range of pH 3.0–3.5 (Supplementary Figure S3). These results are in agreement with the reported optimal pH 3 for oxidation of ABTS, hydroquinone and guaiacol by *Tcu*DyP, and pH 4.5 for oxidation of Reactive Blue 5 by *Asp*DyP2 [36,37]. No activity was detected above pH 7. Then, the steady-state kinetic constants of bacterial *Asp*DyP2 and *Tcu*DyP, and fungal *Aau*DyP, *Per*VPL and *Pch*LiPA oxidizing the five substrates mentioned above at their optimal pH values were estimated, or taken from literature (Table 1). The kinetic constants for reducing substrates determined in this study were measured with 1 mM H_2_O_2_, to ensure saturation conditions.

In the case of the two bacterial DyPs, the highest catalytic efficiency (*k*_cat_/*K*_m_) of *Asp*DyP2 was with RB19 (1300 s^−1^·mM^−1^), followed by Mn^2+^ (540 s^−1^·mM^−1^), DMP (530 s^−1^·mM^−1^) and ABTS (180 s^−1^·mM^−1^). The catalytic efficiencies previously reported for *Asp*DyP2 oxidation of another anthraquinonic dye (Reactive Blue 5) and Mn^2+^ (710 s^−1^·mM^−1^ and 120 s^−1^·mM^−1^, respectively) [36] are in agreement with the current results. In contrast, *Tcu*DyP showed the highest efficiency oxidizing phenolic DMP (1300 s^−1^·mM^−1^), followed by RB19 (200 s^−1^·mM^−1^) and ABTS (180 s^−1^·mM^−1^). The high efficiencies oxidizing RB19 and DMP are due to the high DyP affinity for these compounds. 

In VA oxidation, the catalytic efficiency of *Pch*LiPA was two orders of magnitude higher than estimated for *Per*VPL that, in turn, was approximately another order of magnitude higher than the apparent efficiency of *Aau*DyP (estimated from the slope of the k_obs_ *vs* concentration straight line, since no saturation was observed). These differences are due to the lower VA affinity of *Per*VPL than *Pch*LiPA, and the extremely low affinity of *Aau*DyP, which did not reach saturation conditions. Finally, the two bacterial DyPs were unable to oxidize this nonphenolic aromatic compound under the current standard reaction conditions.

The anthraquinonic RB19 was oxidized by the three DyPs, albeit with different velocities and affinities, the fungal DyP being the most efficient, followed by *Asp*DyP2 and *Tcu*DyP. RB19 is a typical DyP substrate, but it is also oxidized, although less efficiently, by *Per*VPL2 and *Pch*LiPA. In relation to ABTS (2,2′-azino-bis(3-ethylbenzothiazol- ine-6-sulfonic acid) diammonium salt, the enzymes displayed similar catalytic efficiencies (170–300 s^−1^·mM^−1^), except *Aau*DyP with 1800 s^−1^·mM^−1^ and *Per*VPL with 2700 s^−1^·mM^−1^. DMP is well oxidized by DyPs, with *Tcu*DyP showing the highest catalytic efficiency towards this substrate, among all the enzymes analyzed, and *Asp*DyP2 and *Aau*DyP the highest *k*_cat_ values.

Mn^2+^ oxidation was only catalyzed by *Asp*DyP2 and *Per*VPL, with lower activity in the case of the bacterial enzyme. This is often considered an important reaction in lignin degradation, since the Mn^3+^ formed can act at distance from the enzymes, diffusing into the plant cell-wall. Only a few of the DyPs characterized to date oxidize Mn^2+^ efficiently, such as DyP4 from *Pleurotus ostreatus* or the current *Asp*DyP2 [36,47,48]. Other DyPs claimed to oxidize Mn^2+^, such as those from *Rhodococcus jostii* DyPB [49] and *Pseudomonas species* [50,51], always exhibit low catalytic efficiencies. 

### 2.3. Oxidation of Lignin Model Dimers

**Figure 1:**
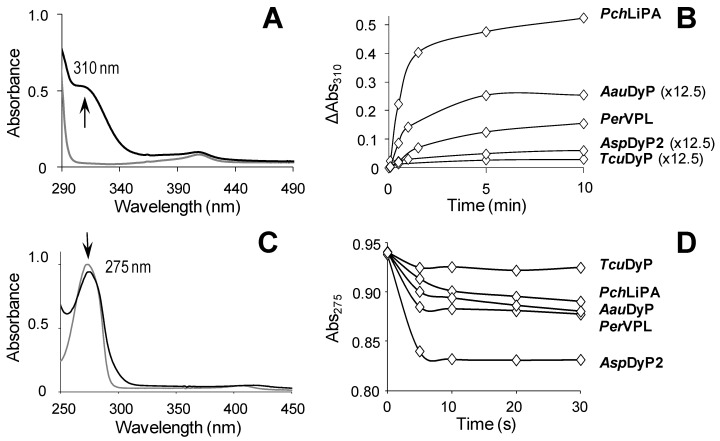
UV-vis analysis of VGE (top) and GGE (bottom) reactions with class-II peroxidases and DyPs. (**A**) *Pch*LiPA reaction with 310-nm increase due to veratraldehyde formation from VGE. (**B**) Increase of 310-nm absorbance during the VGE reactions (using DyP doses 12.5-fold higher than LiP/VP doses). (**C**) *Pch*LiPA reaction with modest 275-nm decrease due to GGE oxidation. (**D**) Decrease of 275-nm absorbance during the GGE reactions. In (**A**) and (**C**), the initial (gray) and final (black) spectra are shown. The VGE (1 mM) reactions included 10 µM DyP plus 1 mM H_2_O_2_, and 0.4 µM class-II peroxidases plus 0.1 mM H_2_O_2_; while the GGE (0.25 mM) reactions included 0.4 µM enzyme plus 0.4 mM H_2_O_2_.

After studying common peroxidase substrates, the capabilities of *Asp*DyP2, *Tcu*DyP, *Aau*DyP, *Per*VPL and *Pch*LiPA to oxidize and degrade lignin were evaluated using alkyl-aryl ether model dimers. In a first approach, the reaction was analyzed by following spectral changes in a diode-array spectrophotometer. The results obtained with the most representative VGE dimer are illustrated in Figure 1A, showing the changes in the whole (290–490 nm) spectrum during the first 10 min of reaction with *Pch*LiPA. The increase of 310-nm absorbance was indicative for the formation of veratraldehyde by C_α_-C_β_ breakdown after nonphenolic dimer oxidation. A 310-nm increase was used to estimate the kinetic constants included below. In the DyP reactions, the enzyme dose was strongly increased (12.5 fold) to confirm the observed lack of VGE oxidation activity. Nevertheless, no veratraldehyde seems to be formed in the bacterial DyP reactions (even at high enzyme concentration), while the highest VGE breakdown would be produced by *Pch*LiPA, followed by *Per*VPL and high-dose (12.5 fold) treatment with *Aau*DyP (Figure 1B). In the reactions with GGE, the spectral changes (250–450 nm) revealed a decrease of the phenolic dimer by all the enzymes analyzed, as shown by the initial decrease of its absorbance at 275 nm (Figure 1C,D). The 275-nm decrease was used to estimate the kinetic constants included below.

**Figure 2:**
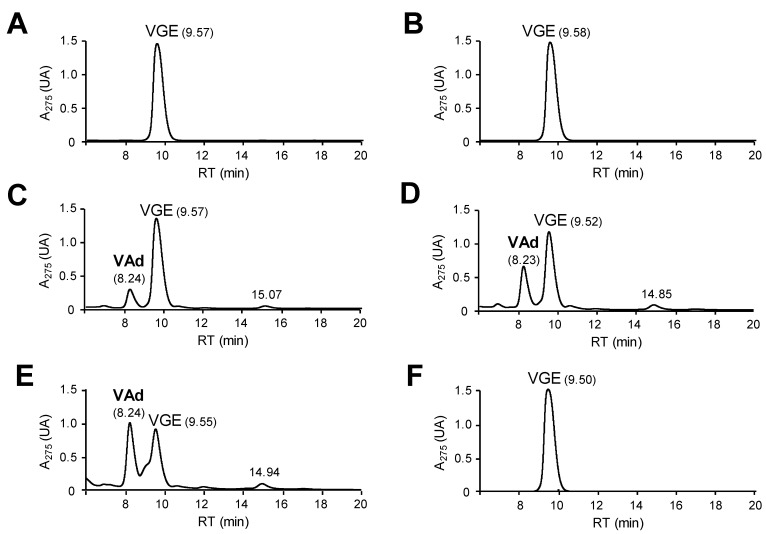
LC analysis of VGE (1 mM) reactions with 4.4 µM *Asp*DyP2 (**A**), *Tcu*DyP (**B**), *Aau*DyP (**C**), *Per*VPL (**D**) and *Pch*LiPA (**E**), and control without enzyme (**F**), in 100 mM tartrate, pH 3, containing 1 mM H_2_O_2_. The reactions were incubated for 1 h at 25 °C and 300 rpm, and stopped with sodium azide before analysis. Veratraldehyde (VAd) formation was observed in (**C**–**E**).

The above spectrophotometric results were confirmed by LC-MS analyses after 1-h reactions of 1 mM VGE with 4.4 µM (Figure 2 and Supplementary Figure S4) and 40 µM (not shown) DyP, VP and LiP doses. The release of veratraldehyde, suggested by the increase of 310-nm absorbance, was confirmed by the chromatographic analysis of the reactions with the fungal enzymes (Figure 2C–E) (based on its retention time and MS spectrum, compared with a true standard), while its absence was confirmed in the reactions with the two bacterial DyPs (Figure 2A,B). In contrast, similar reactions with GGE (Supplementary Figure S5) did not show any breakdown product, and the appearance of a peak with higher molecular mass than GGE suggests that the dimer phenoxy radicals, formed by the action of the enzymes, dimerized [52]. 

To better understand the above results, steady-state kinetic constants of DyP and class-II peroxidase oxidation of phenolic and nonphenolic lignin model dimers were determined, using the absorbance increase at 310 nm, due to veratraldehyde formation from VGE, and the decrease at 275 nm due to GGE disappearance, respectively (initial velocities in both cases). As shown in Table 2, GGE was much more efficiently oxidized than VGE by the different peroxidases (with the only exception of *Pch*LiPA) and the highest GGE catalytic efficiency corresponded to *Asp*DyP2 (with 67 s^−1^·mM^−1^). Moreover, the fungal enzymes oxidized the nonphenolic VGE with catalytic efficiencies that varied from the 14.7 s^−1^·mM^−1^ value of *Pch*LiPA to the only 0.7 s^−1^·mM^−1^ of *Aau*DyP, in contrast to the complete lack of activity of the bacterial DyPs. The kinetic constants for the model dimers were in accordance with those obtained for simple nonphenolic (VA) and phenolic (DMP) aromatics, and the spectrophotometric and LC-MS analyses after longer reaction times with the same dimers, described above.

Our results agree with some previous studies showing that bacterial DyPs, although claimed as lignin-degrading enzymes, are unable to efficiently oxidize and break down nonphenolic lignin model compounds [53]. In this context, the reported 0.086 U·mg^−1^ specific activity for VGE oxidation by *B. subtilis* DyP [26] is more than two orders of magnitude lower than the estimated here for *Pch*LiPA and *Per*VPL (both over 2 U·mg^−1^) and required extremely high (and unrealistic) enzyme doses to see any effect. However, DyPs show good activities towards phenolic lignin model compounds (or kraft lignin that always has high phenolic content). Their GGE specific activities vary from 12–13 U·mg^−1^ reported for the DyPs of *Amycolatopsis* sp and *P. putida* DyP [54] to 1.5 U·mg^−1^ for *Tcu*DyP and only 0.017 U·mg^−1^ for the *R. jostii* DyP [55], with the GGE activities found here for *Pch*LiPA (2.3 U·mg^−1^), *Per*VPL (4.7 U·mg^−1^) and the different DyPs being included in the same activity range. 

### 2.4. Reduction-Potential (E^o^′) Measurements

Enzymes with high reduction-potential are required to oxidize the largely nonphenolic lignin polymer, and the corresponding lignin model dimers. Most of the related literature on class-II and other peroxidases focuses on the heme ferric/ferrous couple, due to easy *E^o^′*(Fe^3+/^Fe^2+^) spectro-electrochemical determination. This includes several bacterial DyPs with *E^o^′*(Fe^3+^/Fe^2+^) values similar to those of generic (i.e., non-ligninolytic) class-II peroxidase of *Coprinopsis cinerea* (between −260 mV and −40 mV for bacterial DyPs, and −183 mV for the fungal generic peroxidase) [36,37,56]. These *E^o^′*(Fe^3+/^Fe^2+^) values are lower than reported for ligninolytic class-II peroxidases (between −137 mV and 50 mV) [57,58]. However, more precise information, including the potential of claimed rate-limiting step in catalytic cycle, corresponding to compound II (CII) to enzyme resting state (RS) reduction, would be required to better understand the ability of DyPs to oxidize lignin related compounds [7].

**Figure 3:**
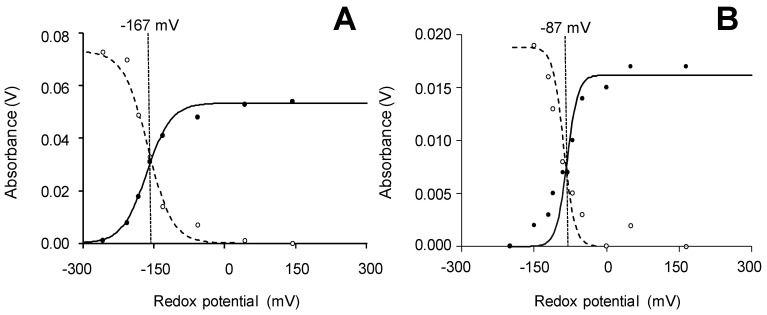
Two examples of spectro-electrochemical titration of Fe^3+^ (continuous line, A_410_) reduction to Fe^2+^ (dashed line, A_438_) in *Aau*DyP (**A**) and *Per*VPL (**B**). The potentials are referred to the standard hydrogen electrode. Data were fitted to the Nernst equation for a reversible one electron transition.

In the present work, we performed classical spectro-electrochemical titration of the Fe^3+^/Fe^2+^ couple, together with equilibrium stopped-flow measurements of the different couples of the catalytic cycle, which provide a more direct picture of the redox properties of the peroxidases analyzed [59,60]. As illustrated in Figure 3A,B (for *Aau*DyP, and *Per*VPL, respectively), the spectro-electrochemical titration follows the Fe^3+^/Fe^2+^ equilibrium through changes of their respective maxima (at 410 and 438 nm) under different applied redox potentials. In this way, *E^o^′*(Fe^3+^/Fe^2+^) values between the −167 mV of *Aau*DyP and the −85 mV of *Asp*DyP2, with intermediate values for *Tcu*DyP (−136 mV), *Pch*LiPA (−90 mV) and *Per*VPL (−87 mV) (standard errors ± 5–10 mV). However, the Fe^3+/^Fe^2+^ couple is not part of the peroxidase catalytic cycle and, therefore, these values are scarcely relevant from an eco-physiological point of view. In this cycle, compound I (CI) formation and reduction to CII are usually fast steps, and the whole reaction would be rate-limited by the slower return to the enzyme RS. Therefore, the *E^o^′* of the later reaction is the key value to predict the ability of different peroxidases to oxidize lignin and other recalcitrant compounds. 

**Figure 4:**
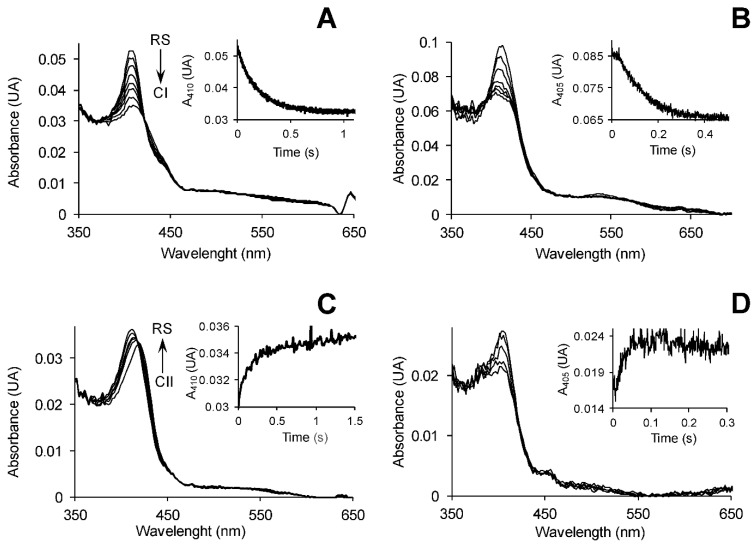
Four examples of stopped-flow measurements for *E^o^′* estimation of peroxidase catalytic couples. (**A**,**B**) Spectral changes during CI formation by H_2_O_2_ addition to *Pch*LiPA and *Asp*DyP2, respectively. (**C**,**D**) Spectral changes upon reduction with tyrosine of the CII species formed by adding H_2_O_2_ and one FeKCN equivalent to *Pch*LiPA and *Asp*DyP2, respectively. The arrows indicate the order of successive spectra from RS to CI and from CII to RS in (**A**/**B**) and (**C**/**D**), respectively. The insets show time traces near the Soret maximum (at 410 nm in (**A**) and (**C**), and 405 nm in (**B**) and (**D**)) to attain equilibrium conditions. All reactions were at optimal pH 3, and 25 °C.

Although the catalytic cycle is spectroscopically atypical in DyPs, we were able for the first time to estimate *E^o^′* values for the three catalytic couples in three of these peroxidases using a stopped-flow procedure [61], recently applied to ligninolytic peroxidases [59]. The *E^o^′* for the CI/RS couple was estimated by following the formation of CI by H_2_O_2_ under stopped-flow conditions, as illustrated in Figure 4A,B for *Pch*LiPA and *Asp*DyP2, respectively (see Supplementary Figure S6 for additional stopped-flow curves). By performing the above reactions in the presence of different initial H_2_O_2_ concentrations, the *E^o^′*(CI/RS) of each peroxidase was estimated from the equilibrium concentrations (as shown in Tables S3–S7). In a similar way, the *E^o^′* of the CII/RS couple was estimated by generating a CII species with one equivalent of FeKCN after H_2_O_2_ addition, and adding tyrosine (Tyr) to return to the RS, as illustrated in Figure 4C,D for *Pch*LiPA, and *Asp*DyP2, respectively (see Supplementary Figure S7 for additional stopped-flow curves). Using different tyrosine concentrations, the *E^o^′*(CII/RS) of each peroxidase was estimated from the equilibrium concentrations attained (as shown in Tables S8–S12). The *E^o^′* for the CI/CII couple in heme peroxidases cannot be experimentally estimated due to the high reactivity and very short lifetime of CI, but it can be inferred from the estimated *E^o^′*(CI/RS) and *E^o^′*(CII/RS) values (as detailed in Section 3) [59]. 

As the *E^o^′* values of the three catalytic couples were compared (Table 3) the highest values in all cases corresponded to the CI/CII couple while the CII/RS values were the lowest ones, and intermediate values were obtained for the two-electron CI/RS reduction, in agreement with results reported for other peroxidases [59,60]. In consequence, the oxidation of lignin and other high redox-potential molecules by these peroxidases will not be limited by the first reductive step in the peroxidase catalytic cycle nor by the initial oxidative step by H_2_O_2_, but by the second reductive step given its lower *E^o^′* values. When the different enzymes were compared (Table 3) the two class-II peroxidases yielded the highest *E^o^′*(CII/RS) values (with 1.330 V for *Per*VPL, and 1.283 V for *Pch*LiPA) followed by *Asp*DyP2 (1.273 V), *Aau*DyP (1.271 V) and *Tcu*DyP (1.232 V). In the latter case, the calculated *E^o^′*(CII/RS) is close to *E^o^′*(Tyr·/Tyr), explaining the scarce spectral changes observed in the stopped-flow experiments with this enzyme. The three former peroxidases (*Per*VPL, *Pch*LiPA and *Asp*DyP2) also showed the highest *E^o^′*(CI/RS) and *E^o^′*(Fe^3+/^Fe^2+^) values. For *Pseudomonas putida* DyP, *E^o^′*(CI/RS) has also been measured by stopped-flow at equilibrium [50] but no information had been reported before on rate-limiting *E^o^′*(CII/RS) in DyPs based on stopped-flow measurements. 

Interestingly, *E^o^′*(CII/RS) of several DyPs, and some other peroxidases, has also been compared by an indirect method based on the use of phenolic compounds with different redox potentials [62]. Using this “phenol oxidation method”, the redox potentials of fungal DyPs from *A. auricula-judae*, *Exidia glandulosa* and *Mycena epipterygia* were reported to vary between 1.10 ± 0.02 and 1.20 ± 0.1 V, which are intermediate values between those for *Pch*LiP (1.26 ± 0.17 V) and phenol-oxidizing soybean (0.93 ± 0.04 V) and *C. cinerea* (1.06 ± 0.07 V) peroxidases estimated simultaneously, but lower than those obtained here using the more reliable stopped-flow procedure.

### 2.5. Surface Features of the Bacterial and Fungal Peroxidases Analyzed

As mentioned above, a major characteristic of lignin is its recalcitrance towards biodegradation, which resulted in the increase of redox-potential of class-II peroxidases during the evolution of ligninolytic fungi [59]. However, lignin is not only a recalcitrant but also a bulky molecule, with an average molecular mass between 10 and >50 kDa after isolation [63]. This characteristic fully hampers its direct interaction with the reactive heme cofactor of peroxidases, which is buried in a central pocket of the protein with limited access from the solvent region. To overcome this second difficulty, a solvent-exposed catalytic tryptophan forming a reactive radical appeared in both LiP and VP enzymes as a convergent evolution case [64]. The tryptophanyl free radical is able to abstract electrons directly from the lignin macromolecule and transfer them to the heme cofactor via an LRET pathway first described in *Pch*LiPA for VA [65] and oligomeric model [66] oxidation, and later in *Per*VPL [44] and other fungal peroxidases [39]. More importantly, it has been recently demonstrated the implication of this tryptophan in the direct oxidation of the lignin macromolecule by a class-II peroxidase (the same *Per*VPL analyzed here) [67,68]. *Trametopsis cervina* LiP is an exception with a tyrosine (instead of tryptophan) as an adduct-forming solvent-exposed catalytic residue [69,70].

**Figure 5:**
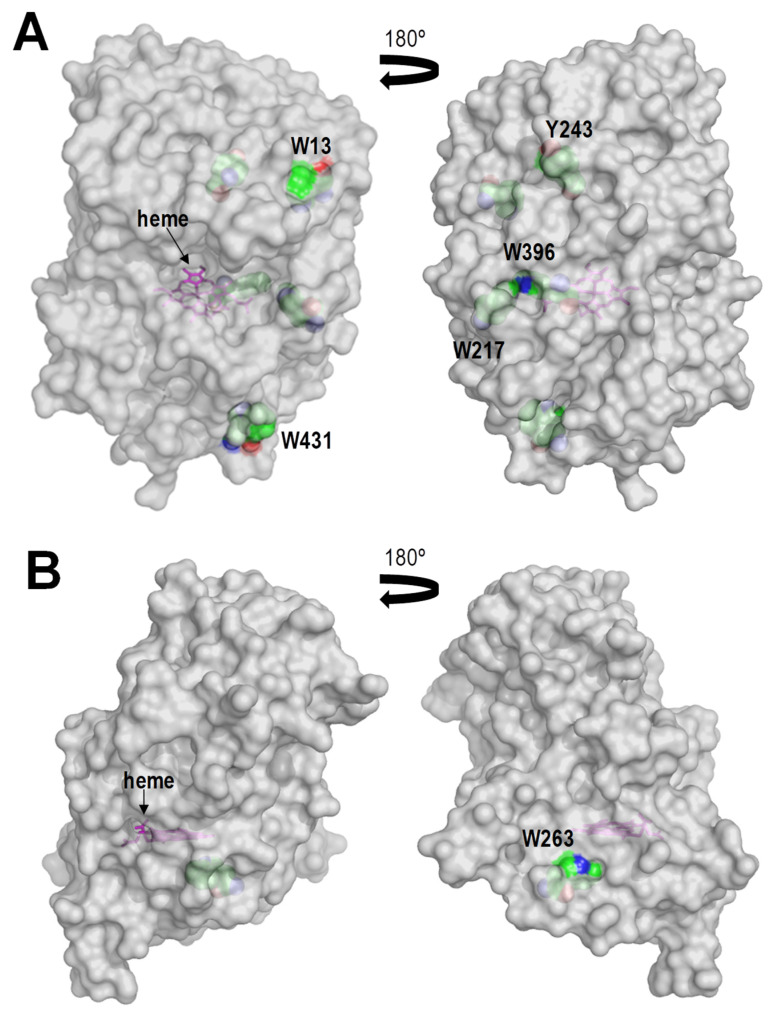
Two views of semi-transparent surfaces of *Asp*DyP2 (**A**) and *Tcu*DyP (**B**) showing the heme (pink sticks) access-channel and several tryptophan and tyrosine residues (CPK-colored van der Waals spheres) located near the protein surface, including *Asp*DyP2 Trp13, Trp217, Trp396, Trp431 and Tyr243, and *Tcu*DyP Trp263.

**Figure 6:**
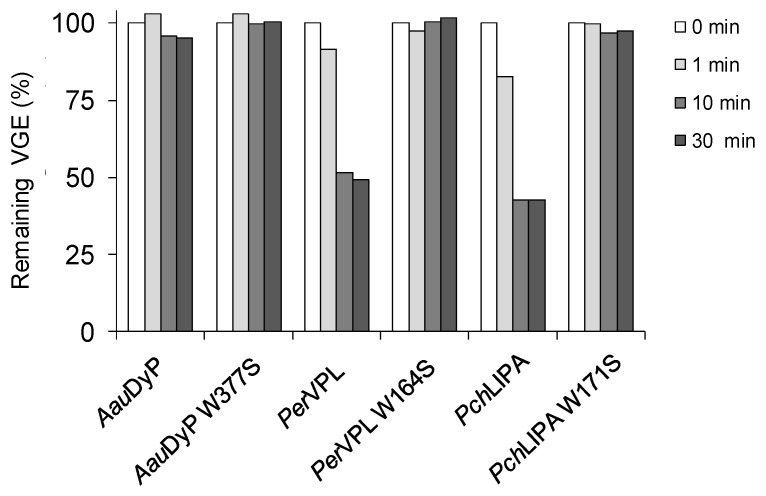
VGE reactions of *Aau*DyP, *Per*VPL, and *PchLiP*A and their tryptophan-less variants. The reactions, including 4.4 µM enzyme and 1 mM VGE in 100 mM tartrate (pH 3), were started by adding 1 mM H_2_O_2_, stopped by sodium-azide addition, and the remaining VGE was quantified at 275 nm after 1, 10 and 30 min and compared with a sample collected before enzyme addition (0 min).

DyPs display in average higher numbers of tryptophan and tyrosine residues than ligninolytic class-II peroxidases, with tyrosine residues being specially rare in the latter enzymes to minimize the risk of inactivation by dimer formation. A total of four tryptophan and seven tyrosine residues exist in the *Aau*DyP mature sequence and three of them (Trp377, Tyr337 and Tyr149) have been reported to be solvent-exposed, including favorable positions for substrate (RB19) oxidation [31,71]. Bacterial DyPs also have high numbers of aromatic amino acids. *Asp*DyP2 has five tryptophan and nine tyrosine residues, with six of them (Trp13, Trp217, Trp396, Trp431, Tyr243 and Tyr353) located near the protein surface, while *Tcu*DyP has seven tryptophan and three tyrosine residues although only Trp263 is located at the vicinity of the surface (Figure 5A,B, respectively). To confirm the involvement of the above tryptophanyl residues in the oxidative degradation of the native (nonphenolic) lignin, Trp-less variants of the three peroxidases degrading VGE (i.e., *Aau*DyP W377S, *Per*VPL W164S and *Pch*LiPA W171S variants) were obtained and evaluated on this model dimer. The results obtained (Figure 6) support that the three enzymes oxidize lignin at this exposed aromatic residue. Although, the effect of its removal is much more evident in the two class-II peroxidases given their stronger action on the VGE dimer. 

**Figure 7:**
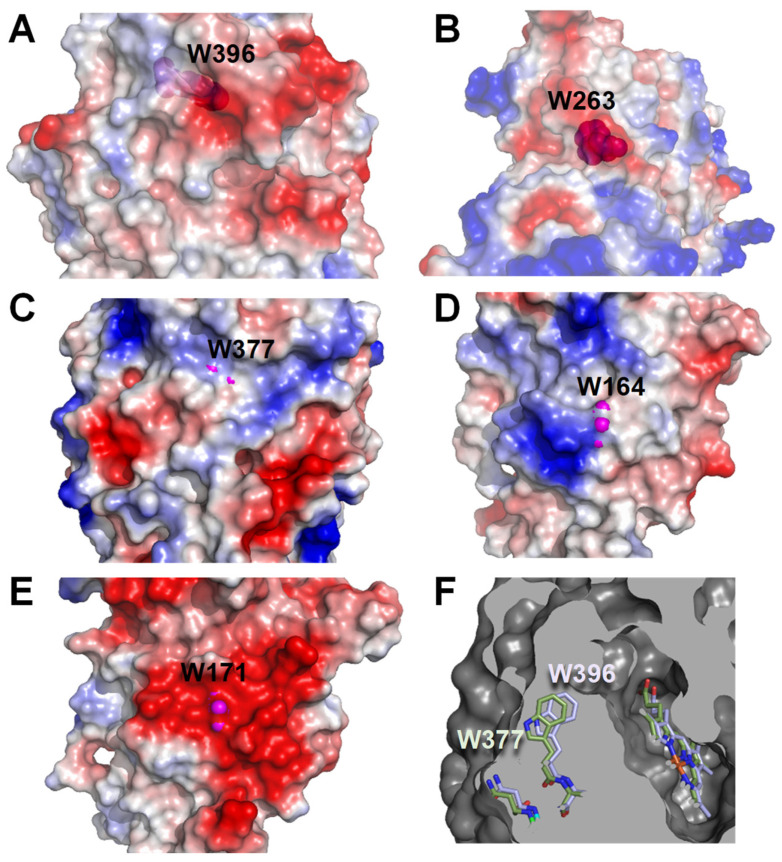
Electrostatic surfaces around the putative catalytic tryptophan of *Asp*DyP2 (**A**), *Tcu*DyP (**B**), *Aau*DyP (**C**), *Per*VPL (**D**) and *Pch*LiPA (**E**), and section comparing the positions of *Asp*DyP2 Trp396 (*blue*) and *Aau*DyP Trp377 (*green*) (**F**). The tryptophan residues are shown as magenta van der Waals spheres, which reach (**C**–**E**) or are located near the solvent ((**A**) and (**B**), with semitransparent surfaces), while they appear as CPK-colored sticks in the surface sections of (**F**).

It had been suggested that an electronegative environment could contribute to the reactivity of the solvent-exposed catalytic tryptophan in ligninolytic peroxidases [46,64]. Therefore, we also analyzed the electrostatic surface surrounding the above tryptophan residues of *Aau*DyP, *Per*VPL and *Pch*LiPA, together with those of *Asp*DyP2 Trp396, homologous to *Aau*DyP Trp377, and *Tcu*DyP Trp263, described as the solvent-exposed catalytic residue in this peroxidase [72]. The strongly-negative Trp171 environment in *Pch*LiPA (Figure 7E) would be in agreement with its ability to efficiently oxidize nonphenolic aromatics [64], but *Per*VPL (Figure 7D) and *Aau*DyP (Figure 7C) do not have such negative environment and they are still able to oxidize them in different extents. Moreover, *Asp*DyP2 (Figure 7A) and *Tcu*DyP (Figure 7B) have partially negative environments, but they are fully inactive on nonphenolic substrates. Taking the above into account, we considered if subtle differences in the position of the tryptophan side-chain could be related to different reactivities by modifying the solvent exposure of the indolic ring. This seems to be the case, since while in *Aau*DyP, *Per*VPL and *Pch*LiPA the side chains are visible from the solvent (magenta spheres Figure 7), in the two bacterial DyPs no significant exposure was observed (note that semitransparent surfaces are included in Figure 7A,B). A more detailed comparison was possible between *Asp*DyP2 and *Aau*DyP because of their structural similarities (both belonging to the DyP type-V). In this way, the *Asp*DyP2 inability to oxidize nonphenolic aromatics can be related to the more-buried nature of the Trp396 side chain, compared with *Aau*DyP Trp377 (Figure 7F), which would hamper electron abstraction from the solvent region. Consequently, the only sites a priori available for catalysis in the two bacterial DyPs would be the heme channel, and the Mn-binding site of *Asp*DyP2 [36]. 

### 2.6. Overview on Relevant Characteristics of Bacterial and Fungal DyPs

During recent years, relevant information on new DyPs has accumulated, including kinetic constants for different substrates, lignin decay assays using phenolic and nonphenolic dimers, some *E^o^′*(Fe^3+^/Fe^2+^) measurements, and several crystal structures. An overview of these DyP characteristic is shown in Table 4 for 14 bacterial DyPs and three fungal DyPs, compared with two class-II peroxidases. All peroxidases analyzed here were able to oxidize phenolic lignin compounds, but only the fungal enzymes oxidize the nonphenolic ones, which are representative for the most abundant substructures in lignin. We also measured for the first time the rate-limiting redox properties of their catalytic states, revealing that the two class-II peroxidases analyzed (*Per*VPL and *Pch*LiPA) have the highest *E^o^′*(CII/RS) values. The latter would be in agreement with their much higher activity on nonphenolic VGE. However, practically no difference was observed between the *E^o^′*(CII/RS) values of *Asp*DyP2 and *Aau*DyP, to explain the absolute lack of VGE activity of *Asp*DyP2 compared with the low, but detectable activity of *Aau*DyP. Nevertheless, we found that differences in the orientation of the catalytic tryptophan side-chain of *Aau*DyP could result in a more exposed tryptophan than in *Asp*DyP2. Therefore, the ability to oxidize the major nonphenolic moiety of lignin by bacterial (and fungal) peroxidases not only requires an adequate redox potential, but it is also probably affected by the side-chain orientation and solvent exposure of a catalytic tryptophan and its charged surface environment. Consequently, given the lack of activity on simple and dimeric lignin model compounds, we cannot assume that bacterial DyPs are ligninolytic enzymes in nature, although they can efficiently oxidize lignin-derived phenols. This activity on phenolic lignin-derived compounds is of interest for the valorization of industrial lignin in the biorefinery context.

## 3. Materials and Methods

### 3.1. Chemicals

Yeast extract was from BD Biosciences (Erembodegem, Belgium); bactotriptone was from Life Technologies Corporation (Detroit, USA); kanamycin, chloramphenicol, dithiothreitol (DTT) and hemin from Sigma-Aldrich (Steinheim, Germany); ABTS and DNase I from Boehringer Mannhein (Mannheim, Germany); GGE from TCI (Zwijdrecht, Belgium); and VGE from Fluorochem (Hadfield, UK). The rest of the compounds used came from Merck (Darmstadt, Germany).

### 3.2. Enzymes Expression, Purification and nLC-MS/MS Analysis

The coding sequences of *Asp*DyP2 from *Amycolatopsis* sp. ATCC 39116 (PDB 4G2C) and *Tcu*DyP from *T. curvata* (PDB 5JXU) were generated in base to the mature protein (excluding the signal peptide in the case of *Tcu*DyP), optimized with OPTIMIZER (http://genomes.urv.es/OPTIMIZER/, accessed on 4 March 2021) for *E. coli* codon usage, synthesized by ATG:biosynthetics and cloned in the expression vector pET28a (conferring kanamycin resistance) incorporating a His tag at the N-terminus. The resulting plasmids, pET28a-*Asp*DyP2 and pET28a-*Tcu*DyP, were introduced into *E. coli* BL21 (DE3) pLysS (exhibiting chloramphenicol resistance). Transformants were selected on Luria-Bertani medium for double resistance to kanamycin and chloramphenicol. For protein production, transformed *E. coli* BL21 strains were grown in 200 mL of the same medium supplemented with kanamycin (30 μg/mL) and chloramphenicol (34 μg/mL) for 18 h at 37 °C and 220 rpm. Thirty mL of this overnight culture were added to 970 mL of self-induction ZYM medium [85] -supplemented with 30 μg/mL kanamycin, 34 μg/mL chloramphenicol and 200 μM hemin- and grown for 4 d at 16 °C and 180 rpm. The cells were pelleted by centrifugation at 8000 rpm for 10 min, frozen at −20 °C, and resuspended in lysis buffer (300 mM NaCl, 20 mM imidazole, 20 mM Tris, pH 8, and 2 mg/mL DNase). After 1-h incubation on ice, the cells were sonicated (3 cycles of 1 min) and centrifuged at 15,000 rpm for 30 min. The supernatants were ultracentrifuged at 36,000 rpm for 90 min, and filtered through 0.22 μm filters (Millipore) before two fast protein liquid chromatography (FPLC) steps using an ÄKTA-purifier (GE Healthcare, Chicago, IL, USA) equipment.

For *Asp*DyP2 purification, a first affinity chromatography was accomplished by loading the cell extract into a nickel-containing HiTrap IMAC column (GE Healthcare). The column was washed with 20 mM imidazole, 500 mM NaCl, 20 mM Tris, pH 8 (solution A), and eluted with a 0–30% gradient of 500 mM imidazole, 500 mM NaCl, 20 mM Tris, pH 8. The *Asp*DyP2 fraction was dialyzed in 10 mM Tris, pH 7.5, loaded into a molecular-exclusion Superdex75 10/300GL column (GE Healthcare), and eluted with an isocratic gradient of 150 mM NaCl in 20 mM Tris, pH 7.4. For *Tcu*DyP purification, the cell extract was also loaded into the above nickel-containing column, washed with solution A and eluted with a 10–40% gradient of 1 M imidazole, 500 mM NaCl, 20 mM Tris, pH 8. The protein fraction was dialyzed in 10 mM Tris, pH 7.5, and injected into an anion-exchange Q-Resource column (GE Healthcare), washed with 20 mM Tris, pH 7.5, and eluted with a 0–30% gradient of 1 M NaCl, 20 mM Tris, pH 7.5. In both purification processes, protein elution was followed at 410 nm (heme proteins) and 280 nm (all proteins). Purification yields were monitored by quantifying proteins at 280 nm using a NanoDrop 2000 (Thermo Scientific, Waltham, MA, USA) equipment, and activity along the different purification steps by measuring 0.5 mM ABTS oxidation in 0.1 M tartrate, pH 3, in the presence of 1 mM H_2_O_2_. Enzyme purity was followed by 12% PAGE in the presence of 0.1% SDS and 1% mercaptoethanol, reducing disulfide bridges and unfolding proteins [86]. Purified *Asp*DyP2 and *Tcu*DyP were dialyzed in 10 mM Tris, pH 7.5, and frozen at −80 °C before use.

Identification of the purified recombinant *Asp*DyP2 and *Tcu*DyP was confirmed by MALDI-TOF/TOF peptide mass fingerprinting. With this purpose, SDS-PAGE bands containing the proteins were digested with trypsin (Thermo, 12.5 ng/µL) in 50 mM ammonium bicarbonate, overnight at 37 °C. After extraction and dying at room temperature, the digested peptides were analyzed with an Autoflex III TOF/TOF mass spectrometer (Bruker-Daltonics, Billerica, MA, USA). Automated analysis of mass data was performed using the FlexAnalysis 3.4 software (Bruker-Daltonics) and comparison with the protein sequences of *Asp*DyP2 and *Tcu*DyP using the MASCOT 2.3 software (Matrix Science). 

Native recombinant *Aau*DyP (GenBankJQ650250, corresponding to isoform I) and its W377S mutated variant were expressed as inclusion bodies in *E. coli* BL21(DE3)pLysS cells, in vitro activated, and purified as described elsewhere [24]. Native *Per*VPL (GenBankAF007222, corresponding to allelic variant-2 of isoenzyme VPL) and its W164S variant, and native *Pch*LiPA (JGI ID# 2989894, corresponding to isoenzyme H8) and its W171S variant, were expressed as inclusion bodies in *E. coli* W3110 cells, in vitro activated, and purified as described elsewhere [40,41].

The pyridine hemochrome assay was used to calculate the concentration of heme in the purified enzymes by adding pyridine to the enzyme reduced with sodium dithionite [87]. Molar extinction coefficients were then calculated according to Beer’s law (A = ε d C; where A is absorbance, d is length of the light beam, and C is molar concentration), using heme concentration and the absorbance at 405 nm of the purified enzymes. 

### 3.3. Enzyme Kinetics

Steady-state kinetics for peroxidase substrates were analyzed spectrophotomet- rically using a Thermo Spectronic UV–visible spectrophotometer in triplicate reactions at 25 °C, after determining the optimal pH for each enzyme and substrate couple. For the latter purpose, oxidation of saturating concentrations of veratryl alcohol (VA, 10 mM), Reactive Blue 19 (RB19, 50 μM), ABTS (500 μM), 2,6-dimethoxyphenol (DMP, 1 mM), and Mn^2+^ (1 mM) were measured in 100 mM Britton-Robinson buffer, at pH between 2 and 9 (triplicate reactions). For the oxidation of VA, DMP, ABTS and Mn^2+^, increases in absorbance at 310 nm (formation of veratraldehyde, ε_310_ = 9300 M^−1^·cm^−1^) 469 nm (formation of dimeric coerulignone, ε_469_ = 55,000 M^−1^·cm^−1^), 436 nm (formation of ABTS cation radical, ε_436_ = 29,300 M^−1^·cm^−1^), and 238 nm (formation of Mn^3+^-tartrate from MnSO_4_, ε_238_ = 6500 M^−1^·cm^−1^) were measured, respectively. For RB19 oxidation, the loss of absorbance (ε_595_ = 10,000 M^−1^·cm^−1^) corresponding to the discoloration of the dye was followed. For each enzyme, the activity at the optimal pH was considered as 100% for comparison. 

Oxidation of phenolic lignin model dimer GGE and nonphenolic VGE was measured over time using a diode-array spectrophotometer Agilent 8453 (Agilent, Santa Clara, CA, USA) in a 200–800 nm wavelength range. The GGE (0.25 mM) plus enzyme (0.4 µM in all cases) and VGE (1 mM) plus enzyme (2.5 µM DyP or 0.2 µM *Per*VPL and *Pch*LiPA) reactions were carried out in 100 mM tartrate, pH 3. After recording the time-zero spectrum, the reactions were started by adding H_2_O_2_ (1 mM for DyP and 0.1 mM for *Per*VPL and *Pch*LiPA in VGE reactions, and 0.4 mM in GGE reactions) and followed for 10 min. The oxidation of GGE was measured by its absorbance loss at 275 nm (ε_275_ = 4400 M^−1^·cm^−1^) while the oxidation of VGE was followed by increase of at 310-nm absorbance, due to veratraldehyde formation. 

Kinetic constants were calculated by oxidation of increasing concentrations of the above enzyme-reducing substrates in 100 mM buffer, at the indicated pH values, using 10 nM enzyme, except for the oxidation of DMP and RB19 with *Tcu*DyP, for which 48 nM enzyme was used, and for the oxidation of GGE and VGE for which higher enzyme doses were required as mentioned above. Kinetic constants for H_2_O_2_ were determined using 0.5 M ABTS as reducing substrate. The kinetic constants for the rest of the substrates were determined using 1 mM H_2_O_2_, which was prepared before use, and its concentration determined at 240 nm (ε_240_ = 39.4 M^−1^·cm^−1^). The curve-fit and data analysis were carried out using SigmaPlot ver. 11.0. The affinity constant (Michaelis-Menten constant, *K_m_*), the turnover number (catalytic constant, *k*_cat_) and its standard errors were obtained by non-linear fit to the Michaelis-Menten model. The catalytic efficiency (*k*_cat_*/K*_m_) and its standard error were calculated by fitting the experimental data to the normalized Michaelis-Menten Equation (1).

(1) $$\;v=\left(\frac{k_{\mathrm{cat}}}{K_{m}}\right)\cdot \left[S\right]/\left(1+\;\frac{\left[S\right]}{K_{m}}\right)$$

### 3.4. Chromatographic Analysis of Model Dimer Oxidation

Enzymatic oxidation of the phenolic GGE and nonphenolic VGE model dimers, by the native peroxidases and their tryptophan-less variants, was further analyzed by liquid chromatography-mass spectrometry (LC-MS). The reaction mixtures containing 1 mM dimer (from a 20 mM solution in ethanol), 40 μM enzyme and 0.6 mM H_2_O_2_ (GGE reactions) or 4.4 μM enzyme and 1 mM H_2_O_2_ (VGE reactions) in 100 mM tartrate, pH 3, were incubated at 25 °C and 300 rpm. Hundred-μL aliquots were taken after 1, 2, 4 and 6 h of incubation and the enzyme was inactivated by adding 5 μL of 10 mM sodium azide before analysis.

The reaction products (and remaining substrate) were analyzed in an LC-MS system (Thermo Mod. FinniganTM LXQTM) equipped with both UV-vis diode-array (Surveyor PDA Plus) and ion-trap (LXQ) detectors, coupled to a Mediterranean-C18 column (4.6 × 150 mm). For elution, three solvents were used in three of the apparatus pumps: (A) 0.1% formic acid, (B) acetonitrile and (C) methanol. For separation of the oxidation products from VGE the employed method included an isocratic gradient of 70% A, 22.5% B and 7.5% C, while for the GGE reaction the mobile phase contained 72% A, 14% B and 14% C. The absorbance was monitored at 230, 275 and 310 nm, while the range of mass tracking was between 100 and 1000 m/z using APCI (atmospheric pressure chemical ionization). One mM standards of VGE, GGE, guaiacol, veratraldehyde and creosol were used. In this way, the retention times of VGE, GGE and some oxidation products, such as veratraldehyde, were determined under the operation conditions. 

### 3.5. Spectro-Electrochemical Measurement of Fe^3+^/Fe^2+^ Redox Potentials

For estimation of *E^o^′*(Fe^3+^/Fe^2+^) of *Asp*DyP2, *Tcu*DyP, *Aau*DyP, *Per*VPL and *Pch*LiPA, electronic absorption spectra, including the characteristic Soret band, were acquired with a Shimadzu UV-2401PC spectrophotometer. Redox titrations of the enzyme samples were controlled with a BAS-CV27 potentiostat and a FLUKE 77 Series II voltmeter. The potentials were calculated *vs* the standard hydrogen electrode at pH 7 (using 20 mM Tris/HCl, with 0.2 M KCl) and 25 °C. For each measurement, 10 µL of enzyme (0.6–1.0 mM concentration) were placed in an ad hoc cell [88] with a 6 µm gold mesh (Buckbee-Mears, Mullheim, Germany) as working electrode, a platinum plate as auxiliary electrode, and a silver/silver chloride reference electrode whose potential was checked prior and after each experiment. The potentiometric titrations were carried out in the presence of 50 µM of the following redox mediators: methylene blue, indigo carmine, 2-hydroxy-1,4- naphtoquinone, anthraquinone-1,5-disulfonate, anthraquinone-2- sulfonate, neutral red and benzyl viologen. To quantify the oxidized and reduced enzyme, we took the absorbance at the 410 nm (Fe^3+^) and 438 nm (Fe^2+^) maxima, and adjusted the values to the Nernst Equations (2) and (3),

(2) $$A_{410}=A_{\max410}\frac{e^{\frac{\left(E\;-\;E^{o\prime }\right)\cdot n\cdot F}{R\cdot T}}}{1+e^{\frac{\left(E\;-\;E^{o\prime }\right)\cdot n\cdot F}{R\cdot T}}}$$

(3) $$A_{438}=A_{\max438}\frac{1}{1+e^{\frac{\left(E\;-\;E^{o\prime }\right)\cdot n\cdot F}{R\cdot T}}}$$

where *R* is 8.31 J·K^−1^·mol^−1^, *T* is set to 298 K, n represents the number of electrons transferred in a single reaction step of the redox couple, and *F* is the Faraday constant with a value of 96,485 J·V^−1^·mol^−1^.

### 3.6. Stopped-Flow Estimation of E^o^′ Values of Catalytic-Cycle Intermediates

We firstly determined (with a comparative purpose) the *E^o^′* of the CI/RS couple in *Asp*DyP2, *Tcu*DyP, *Aau*DyP, *Per*VPL and *Pch*LiPA, corresponding to the enzyme activation by H_2_O_2_. The corresponding values were calculated from equilibrium concentrations estimated by rapid spectrophotometry [59]. RS conversion into CI was followed in a stopped-flow equipment (Bio-Logic, Seyssinet-Pariset, France) synchronized with a diode-array detector (J&M), using the Bio-Kine32 software v4.74.2. The experiments were made in 100 mM tartrate, pH 3, at 25 °C, by mixing enzyme (8 µM DyP and 4 µM VP and LiP) with different concentrations of H_2_O_2_ (1–12 molar equivalents till equilibrium) for 3 s. The *E^o^′*(CI/RS) values were determined using the Nernst equation at equilibrium (4), which correlates the difference of redox potentials between enzyme and substrate with the equilibrium constant K′,

(4) $$\Delta E{{}^{\circ}}^{\prime }=\left(\frac{\mathrm{RT}}{\mathrm{nF}}\right)\cdot l\cdot n\cdot K^{\prime }$$

where *R* is 8.31 J·K^−1^·mol^−1^, *T* is set to 298 K, *n* represents the number of electrons transferred in a single reaction step of the redox couple, and *F* is the Faraday constant with a value of 96,485 J·V^−1^·mol^−1^.

K′ is calculated as indicated in Equation (5):

(5) $$K^{\prime }=\;\frac{\left[H_{2}O_{2}\right]\cdot \left[\mathrm{RS}\right]}{\left[\mathrm{CI}\right]}$$

The amounts of CI and RS were quantified using their extinction coefficients at the equilibrium (i.e., when the spectral changes ended during H_2_O_2_ addition). The RS extinction coefficients values, calculated as explained above, are ε_405_ = 131,100 M^−1^·cm^−1^ for *Asp*DyP2, ε_405_ = 177,500 M^−1^·cm^−1^ for *Tcu*DyP, ε_405_ = 117000 M^−1^·cm^−1^ for *Aau*DyP, ε_410_ = 150,000 M^−1^·cm^−1^ for *Per*VPL, and ε_410_ = 168,000 M^−1^·cm^−1^ for *Pch*LiPA. The CI extinction coefficients were calculated after converting all the RS enzyme into CI using 2–10 H_2_O_2_ equivalents, ensuring there is no self-reduction. The values obtained were ε_405_ = 103,300 M^−1^·cm^−1^ for *Asp*DyP2 CI, ε_405_ = 108,900 M^−1^·cm^−1^ for *Tcu*DyP CI, ε_405_ = 65,750 M^−1^·cm^−1^ for *Aau*DyP CI, ε_410_ = 53,300 M^−1^·cm^−1^ for *Per*VPL CI, and ε_410_ = 111,100 M^−1^·cm^−1^ for *Pch*LiPA CI. 

At a specific wavelength (405 nm for DyPs and 410 nm for *Per*VPL and *Pch*LiPA), the absorbance (*A*_405/410_) is an additive measurement of those of the individual components of a mixture. Therefore, using Soret band extinction coefficients for RS and CI the quantification of the CI/RS redox couple at equilibrium is possible using Equation (6):

(6) $$A_{405/410}=\varepsilon_{405/410-\mathrm{RS}}\cdot \left[\mathrm{RS}\right]\cdot l+{\;\varepsilon }_{405/410-\mathrm{CI}}\cdot \left[\mathrm{CI}\right]\cdot l$$

where l is the path length of the stopped-flow cuvette.

Following a similar procedure, but generating CII with H_2_O_2_ and FeKCN as described below, and using different Tyr concentrations as reducing substrate in stopped-flow equilibrium reactions, we observed different equilibria between the enzyme CII/RS and the Tyr·/Tyr couples, allowing us to apply Equation (4) using the following equilibrium constant:

(7) $$K^{\prime }=\;\frac{\left[\mathrm{Tyr}\cdot \right]\cdot \left[\mathrm{RS}\right]}{\left[\mathrm{Tyr}\right]\cdot \left[\mathrm{CII}\right]}$$

As before mentioned, the concentration of each species is calculated using:

(8) $$A_{405/410}=\varepsilon_{405/410-\mathrm{RS}}\cdot \left[\mathrm{RS}\right]\cdot l+{\;\varepsilon }_{405/410-\mathrm{CII}}\cdot \left[\mathrm{CII}\right]\cdot l$$

To obtain ε_405/410-CII_ we assumed complete CII formation after mixing RS enzyme with one molar equivalent of KFeCN and enough (1–12) equivalents of H_2_O_2_ for 6 s, ensuring no self-reduction to RS enzyme. The different ε_405/410-CII_ values obtained were: ε_405_ = 103,300 M^−1^·cm^−1^ for *Asp*DyP2 CII, ε_405_ = 108,900 M^−1^·cm^−1^ for *Tcu*DyP CII, ε_405_ = 65,750 M^−1^·cm^−1^ for *Aau*DyP CII, ε_410_ = 76,700 M^−1^·cm^−1^ for *Per*VPL CII, and ε_410_ = 105,000 M^−1^·cm^−1^ for *Pch*LiPA CII. All the above reactions were carried out in tartrate 100 mM, pH 3, using *E^o^′*(H_2_O_2_/H_2_O) = 1.56 V and *E^o^′*(Tyr·/Tyr) = 1.18 V values in the Nerst equation.

Finally, assuming the classical catalytic cycle of heme-peroxidases going from RS enzyme to CII via CI with two consecutive one-electron oxidations of substrates, we could deduce the *E^o^′*(CI/CII) values taking into account that the free energy of the reaction (G) and the *E^o^′* are related:

(9) $$\Delta Gr{{}^{\circ}}^{\prime }=-n\cdot F\cdot E{{}^{\circ}}^{\prime }$$

where *n* = 2 electrons for the reduction of CI to RS, Δ*G*r^o^′ equals to −2 · F · [*E^o^′*(CI/RS)] being the sum of the reaction free energy of one electron reductions of CI to CII, and CII to RS.

Therefore, the sum of reaction free energies Δ*G*r^o^′(CI/CII) + Δ*G*r^o^′(CII/RS) and the experimentally-determined *E^o^′*(CII/RS) allows calculation of *E^o^′*(CI/CII).

### 3.7. Structural Analysis of the Different Peroxidases

To investigate the different reactivity towards lignin dimers, we analyze the surface surrounding the solvent-exposed catalytic residue in the DyP, VP and LiP enzymes analyzed. Using PyMol Molecular Graphics Systems (version 2.4.1, Schrödinger LLC) and SwissPDB viewer (version 4.1.0, Swiss Institute of Bioinformatics), we aligned the structures corresponding to *Asp*DyP2 (4g2c), *Tcu*DyP (5jxu), *Aau*DyP (4w7j), *Per*VPL2 (2boq), and *Pch*LiPA (1b82). The electrostatic surfaces were computed using the default parameters in PyMol. Moreover, the position of the catalytic tryptophan side-chain in the *Asp*DyP2 and *Aau*DyP molecular structures was compared after alignment. 

## 4. Conclusions

Studies on lignin biodegradation are important for both understanding the natural cycling of the carbon fixed by land photosynthesis, and developing biotechnological tools for valorizing all biomass constituents in lignocellulose biorefineries to implement a sustainable bioeconomy. Dye-decolorizing peroxidases (DyPs) are an old enzyme family shared by archaea, bacteria and fungi. Its involvement in different stages of lignin biotransformation was not well-established, due to the partially-contradictory information present in the literature. Here, we contribute to establishing the biodegradative capabilities of fungal and bacterial DyPs by comparing their kinetic constants, *E^o^′* values, and relevant structural properties with those of the well-known ligninolytic peroxidases from basidiomycetes. The results revealed that only the fungal enzymes are able to oxidize nonphenolic model dimers, representative for the main moiety of the lignin macromolecule, in parallel with their higher *E^o^′* values. Simultaneously, the activity of bacterial DyPs on phenolic dimeric and simple aromatics suggests that they contribute to the catabolism of lignin products from the fungal attack to native lignin. Therefore, they would be of interest in the biotransformation of technical lignins from industrial operation (e.g., in paper pulp and bioethanol production) since these lignins are often characterized by their high phenolic contents. 

## Figures and Tables

**Table 1:** Steady-state kinetic parameters −*K*_m_ (µM), *k*_cat_ (s^−1^) and *k*_cat_/*K*_m_ (s^−1^·mM^−1^)−for ABTS, DMP, RB19, Mn^2+^, VA and H_2_O_2_ reactions of the bacterial *Asp*DyP2 and *Tcu*DyP, obtained here, compared with fungal *Aau*DyP, *Per*VPL and *Pch*LiPA from previous work.

		ABTS	DMP	RB19	Mn^2+^	VA	H_2_O_2_^1^
*Asp*DyP2	*K*_m_	165 ± 18	177 ± 34	20 ± 4	78 ± 18	-	42 ± 9
	*k*_cat_	30.0 ± 0.9	94.0 ± 4.6	26.0 ± 2.0	42.0 ± 4.0	0	46.0 ± 4.6
	*k*_cat_ */K*_m_	180 ± 20	530 ± 80	1300 ± 200	540 ± 80	-	1100 ± 100
*Tcu*DyP	*K*_m_	309 ± 95	2 ± 0.3	7 ± 2	-	-	157 ± 29
	*k*_cat_	53.0 ± 6.1	2.7 ± 0.4	1.3 ± 0.1	0	0	59.0 ± 1.2
	*k*_cat_ */K*_m_	170 ± 30	1300 ± 100	200 ± 40	-	-	380 ± 10
*Aau*DyP ^2^	*K*_m_	123 ± 7	703 ± 60	90 ± 10	-	-	137 ± 26
	*k*_cat_	225.0 ± 3.0	120.0 ± 3.0	224.0 ± 10.0	0	0	223.0 ± 23.0
	*k*_cat_ */K*_m_	1800 ± 90	173 ± 22	2400 ± 180	-	0.1 ± 0	1600 ± 200
*Per*VPL ^3^	*K*_m_	3 ± 0.2	78 ± 8	33 ± 8	181 ± 10	4130 ± 320	150 ± 16
	*k*_cat_	8.1 ± 0.2	5.6 ± 0.1	8.5 ± 0.7	275.0 ± 4.0	9.5 ± 0.2	36 ± 1
	*k*_cat_ */K*_m_	2700 ± 140	71 ± 6	260 ± 60	1520 ± 70	2 ± 0.1	241 ± 17
*Pch*LiPA ^4^	*K*_m_	21 ± 2	7 ± 1	34 ± 13	-	79 ± 18	82 ± 4
	*k*_cat_	6.5 ± 0.2	4.0 ± 0.1	1.5 ± 0.3	0	16.2 ± 0.8	35 ± 1
	*k*_cat_ */K*_m_	300 ± 25	600 ± 36	45 ± 20	-	205 ± 4	420 ± 16

^1^ Using 0.5 mM ABTS (0.1 mM for *Per*VPL) as reducing substrate. ^2^ DMP and RB19 values of high-turnover site (at Trp377) [24]. ^3,4^ ABTS and DMP values of high-efficiency site, corresponding to *Per*VPL Trp164 [44,45] and *Pch*LiPA Trp171 [46], respectively. All measurements were made at the optimum pH: pH 5 for Mn^2+^ oxidation by *Per*VPL and *Asp*DyP2, and ABTS oxidation by *Asp*DyP2; pH 4 for DMP oxidation by *Asp*DyP2; pH 3.5 for ABTS and DMP oxidation by *Per*VPL and *Pch*LiPA; pH 3 for VA oxidation by *Per*VPL and *Pch*LiPA, DMP oxidation by *Pch*LiPA, *Aau*DyP and *Tcu*DyP, and ABTS oxidation by *Aau*DyP and *Tcu*DyP; and pH 2.5 for VA oxidation by *Aau*DyP.

**Table 2:** Steady-state kinetic parameters −*K*_m_ (µM), *k*_cat_ (s^−1^) and *k*_cat_/*K*_m_ (s^−1^·mM^−1^)− for the oxidation of GGE and VGE lignin model dimers by bacterial *Asp*DyP2 and *Tcu*DyP and fungal *Aau*DyP, *Per*VPL and *Pch*LiPA.

	Phenolic GGE			Nonphenolic VGE		
	*K*_m_	*k*_cat_	*k*_cat_/*K*_m_	*K*_m_	*k*_cat_	*k*_cat_/*K*_m_
*Asp*DyP2	179 ± 39	12.0 ± 1.2	67.0 ± 8.5	-	0	-
*Tcu*DyP	80 ± 16	1.1 ± 0.1	14.2 ± 1.8	-	0	-
*Aau*DyP	-	0	19.8 ± 1.1 ^1^	448 ± 59	0.3 ± 0.01	0.7 ± 0.1
*Per*VPL	206 ± 17	2.7 ± 0.1	13.2 ± 0.7	659 ± 80	1.2 ± 0.1	1.8 ± 0.1
*Pch*LiPA	208 ± 41	1.3 ± 0.1	6.5 ± 0.7	88 ± 3	1.3 ± 0.2	14.7 ± 0.4

^1^ Only apparent *k*_cat_/*K*_m_ value, from linear adjustment, since enzyme saturation was not be attained. All measurements were made in 100 mM tartrate, pH 3, with 1 mM H_2_O_2_.

**Table 3:** *E^o^’* values (in V) of the CI/RS, CI/CII and CII/RS couples, estimated from stopped-flow experiments, in bacterial *Asp*DyP2 and *Tcu*DyP, and fungal *Asp*DyP2, *Per*VPL and *Pch*LiPA.

	*E^o^’*(CI/RS)	*E^o^’*(CI/CII) ^1^	*E^o^’*(CII/RS) ^2^
*Asp*DyP2	1.423 ± 0.002	1.573 ± 0.009	1.273 ± 0.013
*Tcu*DyP	1.371 ± 0.004	1.510 ± 0.002	1.232 ± 0.010
*Aau*DyP	1.368 ± 0.004	1.465 ± 0.005	1.271 ± 0.003
*Per*VPL	1.383 ± 0.004	1.436 ± 0.003	1.330 ± 0.011
*Pch*LiPA	1.402 ± 0.002	1.520 ± 0.006	1.283 ± 0.007

^1^ Deduced from the *E^o^’*(CI/RS) and *E^o^’*(CII/RS) values. ^2^ In contrast with class-II peroxidases, typical CI and CII were not observed during spectrophotometric exploration of the DyP cycle, but the redox species obtained by their treatment with H_2_O_2_ followed by 1 equiv of FeKCN (as used with *Per*VPL and *Pch*LiPA) behave as CII during their return to the RS in the presence of different tyrosine concentrations, allowing *E^o^’* estimation.

**Table 4:** Comparison of catalytic efficiencies (s^−1^·M^−1^) oxidizing RB19, DMP, VA and Mn^2+^, lignin decay assays (including action on GGE and VGE dimers), and Fe^3+^/Fe^2+^ and CII/RS reduction potentials (in V) of 14 bacterial and 3 fungal DyPs compared with LiP and VP (brackets correspond to the present study). PDB crystal structures are also indicated. nd, not determined; tr, traces.

Type	Organism	RB19	DMP	VA	Mn^2+^	Lignin Decay Assays	Fe^3+^/Fe^2+^	CII/RS	PDB	Refs
DyP-I	*Bacillus subtilis*	+	nd	tr	nd	Good GGE (very low VGE) oxidation (HPLC)	−0.040	nd	nd	[26,56]
DyP-I	*Thermobifida fusca*	+	+	tr	0	Guaiacol, kraft lignin, GGE (no VGE) oxidation	nd	nd	5fw4	[42,52,53]
DyP-I	*Thermomonospora curvata*	2300–7800 · 10^3^	[1.3 · 103]	[0]	[0]	GGE [no VGE] oxidation [HPLC, kinetics] MeO-mandelic acid oxidation	[−0.136]	[1.232]	5jxu	[37,72] [this study]
DyP-P	*Enterobacter lignolyticus*	4200 · 10^3^	nd	0	tr	Low oxidation of phenols (no VA) but good dye oxidation	−0.290	nd	5vj0	[73]
DyP-P	*Escherichia coli*	+	nd	nd	nd	Oxidation of phenols	nd	nd	5gt2	[74]
DyP-P	*Klebsiella pneumoniae*	nd	nd	nd	nd	nd	−0.350	nd	6fks	[75]
DyP-P	*Pseudomonas fluorescens* (DyP1B)	nd	0.53 · 10^3^	nd	0.33–0.66 · 103	Kraft lignin (465 nm; with kcat/Km 140 · 103) wheat straw (HPLC)	nd	nd	nd	[51,76]
DyP-P	*Pseudomonas putida*	+	0.7 · 10^3^	tr	52 · 10^3^	Kraft lignin (LC-MS) GGE (HPLC)	−0.260	nd	nd	[54,77,78,79]
DyP-P	*Pseudomonas* sp	nd	9.3 · 10^3^	nd	34 · 10^3^	Guaiacol oxidation, phenols from lignin	nd	nd	nd	[19]
DyP-P	*Rhodococcus jostii* (DyPB)	nd	nd	nd	0.025 · 10^3^	Nitrated lignin assay, kraft lignin, GGE (HPLC, stopped flow) oxidation	nd	nd	3veg	[49,55]
DyP-P	*Vibrio cholerae*	26 · 10^3^	nd	nd	nd	Guaiacol oxidation	nd	nd	5de0	[80]
DyP-V	*Anabaena* sp	+	nd	nd	nd	Oxidation of phenols	nd	nd	5c2i	[27]
DyP-V	*Amycolatopsis* sp (DyP2)	[1300 · 103]	[0.53 · 103]	[0]	[0.54]–120 · 103	[GGE (no VGE) oxidation (HPLC, kinetics)], MeO-mandelic oxidation	[−0.085]	[1.273]	4g2c	[36] [this study]
DyP-V	*Streptomyces avermitilis*	nd	0.067 · 10^3^	nd	nd	Anthraquinonic dye oxidation	nd	nd	nd	[81]
DyP-V	*Auricularia auricula-judae*	4800 · 103	3900–2000 · 103	0.2–0.11 · 103	0	[GGE and VGE (low) (HPLC, kinetics)] other nonphenolic dimers (low)	[−0.160]	[1.271]	4au9 4w7j	[24,38] [this study]
DyP-V	*Irpex lacteus*	5900 · 10^3^	970 · 10^3^	0.83 · 10^3^	0	Increased digestibility of treated wheat straw	nd	nd	nd	[82]
DyP-V	*Pleurotus ostreatus* (DyP4)	1860 · 10^3^	2120 · 10^3^	0	196 · 10^3^	Phenolic lignin oxidation given Mn-oxidizing activity	nd	nd	6fsk	[48]
LiP	*Phanerochaete chrysosporium* (LiPA)	[0.045 · 103]	1720 · 103	92 · 103	0	Lignosulfonate transient-state kinetics and NMR [GGE and VGE (HPLC, kinetics)]	[−0.090]	[1.330]	1qpa	[83,84] [this study]
VP	*Pleurotus eryngii* (VPL)	[0.26 · 103]	71 · 103	2.3 · 103	1520 · 103	Lignosulfonate transient-state kinetics and NMR [GGE and VGE (HPLC, kinetics)]	[−0.087]	[1.283]	2boq	[67,68,83] [this study]

## Data Availability

The raw data supporting the conclusions of this article will be made available by the authors, without undue reservation, or are already included in the main manuscript and its Supplementary Material.

## Supplementary Materials

Supplementary data to this article are available online at https://www.mdpi.com/1422-0067/22/5/2629/s1.
