# Supplementary material for: Comparing Ligninolytic Capabilities of Bacterial and Fungal Dye-Decolorizing Peroxidases and Class-II Peroxidase-Catalases
Source: Int J Mol Sci. 2021 Mar 5;22(5):2629. doi: 10.3390/ijms22052629 (PMC7961821; doi:10.3390/ijms22052629)
Supplement: Supplementary file 1 [file ijms-22-02629-s001.pdf]

SUPPLEMENTARY INFORMATION

Comparing Ligninolytic Capabilities of Bacterial and Fungal Dye-Decolorizing Peroxidases and Class-II Peroxidase-Catalases

Dolores Linde,[1‡] Iván Ayuso-Fernández,[1‡#] Marcos Laloux,[1] José E. Aguiar-Cervera,[1] Antonio L. de Lacey,[2] Francisco J. Ruiz-Dueñas,[1] Angel T. Martínez[1*]

[1] Centro de Investigaciones Biológicas "Margarita Salas" (CIB), CSIC, Ramiro de Maeztu 9, E-28040, Madrid, Spain; lolalinde@cib.csic.es (DL), ivan.ayuso-fernandez@nmbu.no (IA-F), marcos.laloux@gmail.com (ML), joseac93@hotmail.com (JEA) and fjruiz@cib.csic.es (FJR-D)
[2] Instituto de Catálisis y Petroleoquímica (ICP), CSIC, Marie Curie 2, E-28049 Madrid, Spain; alopez@icp.csic.es (ALL)
[*] Correspondence: atmartinez@cib.csic.es (ATM)
[‡] These two authors contributed equally to this work
[#] Current address: Norwegian University of Life Sciences (NMBU), Ås, Norway

This supplementary information includes: Summary of the purification processes (**Tables S1** and **S2**); CI/RS equilibrium concentrations and $E^{o\prime}$(CI/RS) values (**Tables S3-S7**); CII/RS equilibrium concentrations and $E^{o\prime}$(CII/RS) values (**Tables S8-S12**); chemical structures of lignin model dimers (**Figure S1**); SDS-PAGE from the purification processes (**Figure S2**); optimal pH for the different reactions (**Figure S3**), LC analyses of model-dimer reactions (**Figure S4** and **S5**); spectral changes during CI formation (**Figure S6**); and spectral changes upon reduction with tyrosine of CII (**Figure S7**).

TABLE S1. Summary of recombinant *Asp*DyP2 purification process from 5-L *E. coli* culture

	Protein (mg)	Total activity (U)	Specific activity (U/mg)	Yield (%)	Purification factor
Cell extract	3770	98.1	0. 03	100	1
Affinity chromatography	1.4	32.1	21.5	32.4	716
Mw-exclusion chromatography	0.5	9.7	19.4	9.5	650

Activity measured with 0.5 mM ABTS in 0.1 M tartrate, pH 3, in the presence of 1 mM H_2O_2

TABLE S2. Summary of recombinant *Tcu*DyP purification process from 5-L *E. coli* culture

	Protein (mg)	Total activity (U)	Specific activity (U/mg)	Yield (%)	Purification factor
Cell extract	5140	503	0.1	100	1
Affinity chromatography	3.9	22	5.6	4.4	56
Anion-exchange chromatography	0.6	14	23	2.7	230

Activity measured with 0.5 mM ABTS in 0.1 M tartrate, pH 3, in the presence of 1 mM H_2O_2

TABLE S3. Parameters of redox equilibrium and calculated $E^{o'}$ of the CI/RS redox couple of *Asp*DyP2, as a function of the initial concentration of H_2O_2 (all reactions at optimal pH 3).

Initial H_2O_2	Equilibrium concentrations (μM)			$E^{o'}$ (V)
(μM)	*Asp*DyP2-CI	*Asp*DyP2-RS	H_2O_2	
20.00	0.98	3.37	19.02	1.436
40.00	2.90	1.45	37.10	1.420
80.00	3.81	0.54	76.19	1.413
Mean and 95% confidence interval:				1.423 ± 0.002

TABLE S4. Parameters of redox equilibrium and calculated $E^{o'}$ of the CI/RS redox couple of *Tcu*DyP, as a function of the initial concentration of H_2O_2 (all reactions at optimal pH 3).

Initial H_2O_2	Equilibrium concentrations (μM)			$E^{o'}$ (V)
(μM)	*Tcu*DyP-CI	*Tcu*DyP-RS	H_2O_2	
4.00	2.65	0.60	1.347	1.367
6.00	2.90	0.35	3.10	1.370
8.00	3.00	0.25	5.00	1.372
10.00	3.00	0.25	7.00	1.376
Mean and 95% confidence interval:				1.371 ± 0.004

TABLE S5. Parameters of redox equilibrium and calculated $E^{o'}$ of the CI/RS redox couple of *Aau*DyP, as a function of the initial concentration of H_2O_2 (all reactions at optimal pH 3).

Initial H_2O_2	Equilibrium concentrations (μM)			$E^{o'}$ (V)
(μM)	*Aau*DyP-CI	*Aau*DyP-RS	H_2O_2	
2.00	1.70	2.70	0.30	1.368
4.00	2.96	1.44	1.04	1.373
6.00	3.95	0.45	2.05	1.363
8.00	4.13	0.27	3.87	1.365
Mean and 95% confidence interval:				1.368 ± 0.004

TABLE S6. Parameters of redox equilibrium and calculated $E^{o\prime}$ of the CI/RS redox couple of *Per*VPL, as a function of the initial concentration of H_2O_2 (all reactions at optimal pH 3).

Initial H_2O_2	Equilibrium concentrations (μM)			$E^{o\prime}$ (V)
(μM)	*Per*VPL-CI	*Per*VPL-RS	H_2O_2	
0.50	0.34	1.76	0.16	1.381
1.00	0.56	1.54	0.44	1.385
2.00	0.90	1.20	1.10	1.388
3.00	1.39	0.71	1.61	1.380
Mean and 95% confidence interval:				1.383 ± 0.004

TABLE S7. Parameters of redox equilibrium and calculated $E^{o\prime}$ of the CI/RS redox couple of *Pch*LiPA as a function of the initial concentration of H_2O_2 (all reactions at optimal pH 3).

Initial H_2O_2	Equilibrium concentrations (μM)			$E^{o\prime}$ (V)
(μM)	*Pch*-LiPA-CI	*Pch*-LiPA-RS	H_2O_2	
1.50	0.27	1.97	1.23	1.411
2.00	0.41	1.85	1.59	1.408
3.00	0.98	1.28	2.02	1.395
4.00	1.14	1.13	2.86	1.396
8.00	1.53	0.73	6.47	1.399
Mean and 95% confidence interval:				1.402 ± 0.002

TABLE S8. Parameters of redox equilibrium and calculated $E^{o\prime}$ of the CII/RS redox couple of *Asp*DyP2 as a function of the initial concentration of tyrosine (all reactions at optimal pH 3).

Initial Tyr	Equilibrium concentrations (μM)				$E^{o\prime}$ (V)
(μM)	*Asp*DyP2-RS	*Asp*DyP2-CII	Tyr	Tyr·	
5.00	0.36	1.12	4.64	0.36	1.271
10.00	0.48	1.00	9.52	0.48	1.273
25.00	0.55	0.93	24.45	0.55	1.287
50.00	0.98	0.50	49.02	0.98	1.259
Mean and 95% confidence interval:					1.273 ± 0.013

TABLE S9. Parameters of redox equilibrium and calculated $E^{o\prime}$ of the CII/RS redox couple of *Tcu*DyP as a function of the initial concentration of tyrosine (all reactions at optimal pH 3).

Initial Tyr	Equilibrium concentrations (μM)				$E^{o\prime}$ (V)
(μM)	*Tcu*DyP-RS	*Tcu*DyP-CII	Tyr	Tyr·	
5.00	0.39	0.47	4.61	0.39	1.245
20.00	0.70	0.16	19.30	0.70	1.223
50.00	0.75	0.11	49.25	0.75	1.229
100.00	0.79	0.07	99.21	0.79	1.232
Mean and 95% confidence interval:					1.232 ± 0.010

TABLE S10. Parameters of redox equilibrium and calculated $E^{o\prime}$ of the CII/RS redox couple of *Aau*DyP as a function of the initial concentration of tyrosine (all reactions at optimal pH 3).

Initial Tyr	Equilibrium concentrations (μM)				$E^{o\prime}$ (V)
(μM)	*Aau*DyP-RS	*Aau*DyP-CII	Tyr	Tyr·	
5.00	0.24	0.56	4.76	0.24	1.275
10.00	0.33	0.46	9.67	0.33	1.271
20.00	0.43	0.37	19.57	0.43	1.270
25.00	0.47	0.33	24.53	0.47	1.268
Mean and 95% confidence interval:					1.271 ± 0.003

TABLE S11. Parameters of redox equilibrium and calculated $E^{o\prime}$ of the CII/RS redox couple of *Per*VPL as a function of the initial concentration of tyrosine (all reactions at optimal pH 3).

Initial Tyr	Equilibrium concentrations (μM)				$E^{o\prime}$ (V)
(μM)	*Per*VPL-RS	*Per*VPL-CII	Tyr	Tyr·	
20.00	0.22	1.88	19.78	0.22	1.347
50.00	0.51	1.59	49.49	0.51	1.323
100.00	0.68	1.42	99.32	0.68	1.323
150.00	0.76	1.34	149.24	0.76	1.326
Mean and 95% confidence interval:					1.330 ± 0.011

TABLE S12. Parameters of redox equilibrium and calculated $E^{o\prime}$ of the CII/RS redox couple of *Pch*-LiPA as a function of the initial concentration of tyrosine (all reactions at optimal pH 3).

Initial Tyr	Equilibrium concentrations (μM)				$E^{o\prime}$ (V)
(μM)	*Pch*LiPA-RS	*Pch*LiPA-CII	Tyr	Tyr·	
10.00	0.42	1.50	9.58	0.42	1.289
20.00	0.62	1.30	19.38	0.62	1.283
50.00	0.95	0.97	49.05	0.95	1.279
100.00	1.19	0.73	98.81	1.19	1.277
Mean and 95% confidence interval:					1.284 ± 0.006

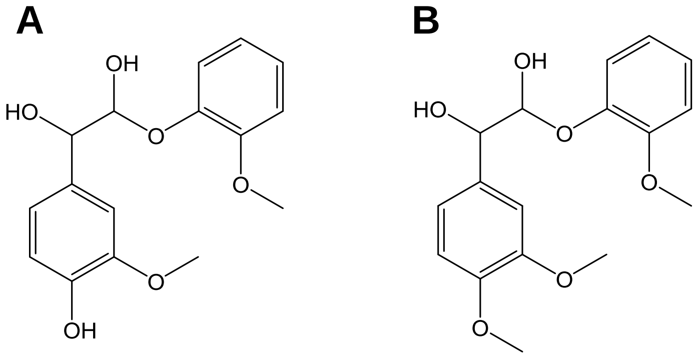

FIGURE S1. Chemical structures of lignin model dimers. (**A**) Guaiacylglycerol-β-guaiacyl ether (GGE). (**B**) Veratryl glycerol-β-guaiacyl ether (VGE).

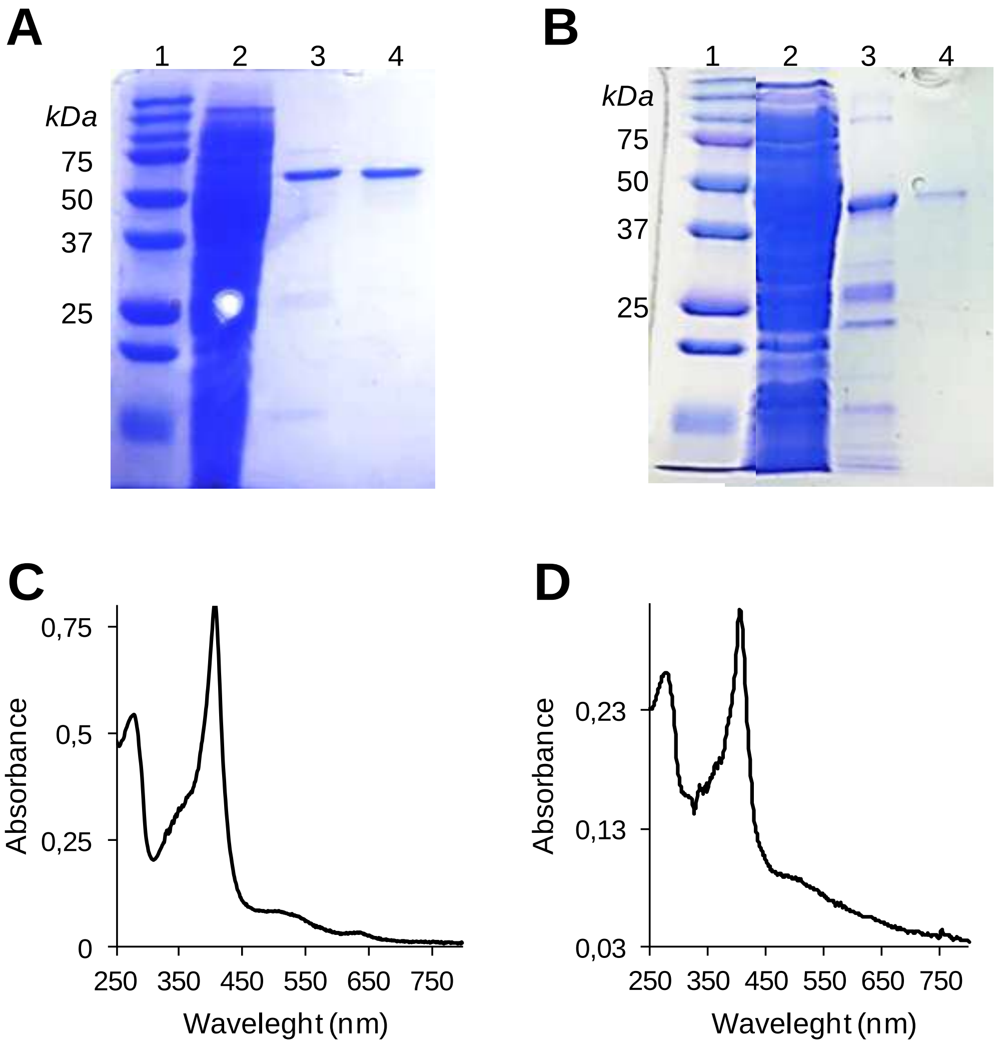

FIGURE S2. SDS-PAGE and electronic absorption spectra from enzyme purification. **A**) SDS-PAGE from *Asp*DyP2 purification including *E. coli* cell extract (*lane 2*), protein fraction after Ni-affinity chromatography (*lane 3*), pure *Asp*DyP2 after molecular-exclusion chromatography (*lane 4*), and molecular-mass standards (*lane 1*). **B**) SDS-PAGE from *Tcu*DyP purification including *E. coli* cell extract (*lane 2*), protein fraction after Ni-affinity chromatography (*lane 3*), pure *Tcu*DyP after anion-exchange chromatography (*lane 4*), and molecular-mass standards (*lane 1*). **C** and **D**) Electronic absorption spectra of pure *Asp*DyP2 and *Tcu*DyP, respectively, after dialysis in Tris, pH 7.

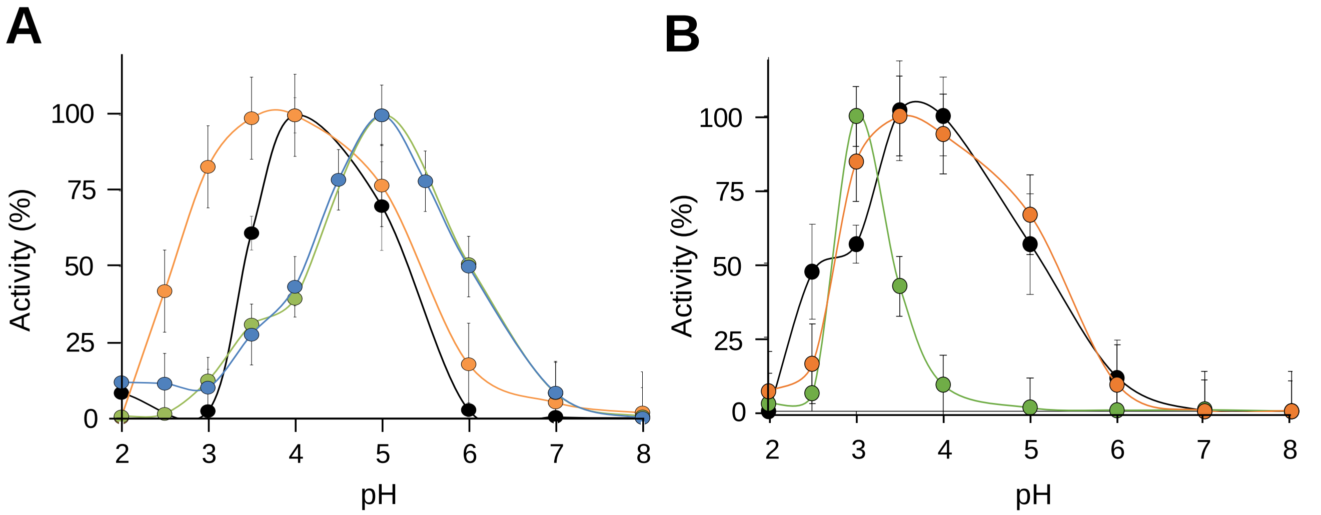

FIGURE S3. Optimal pH of *Asp*DyP2 (**A**) and *Tcu*DyP (**B**) oxidizing ABTS (*green*), RB19 (*black*) DMP (*orange*) and Mn^{2+} (*blue*). Relative activities (%) are referred to the maximal activity on each substrate.

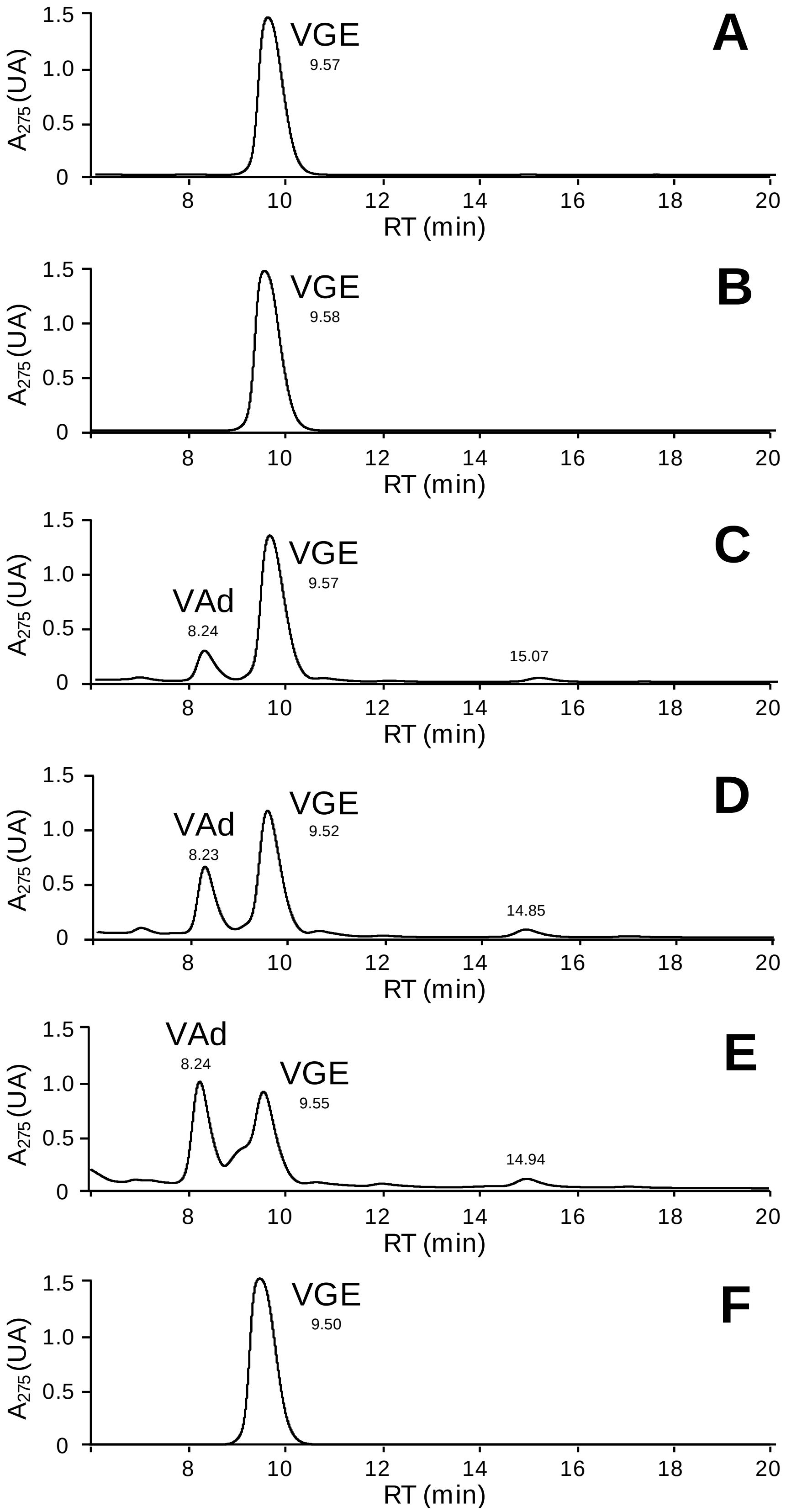

FIGURE S4. LC analysis (275-nm profiles) of VGE (1 mM) reactions with 4.4 μM *Asp*DyP2 (**A**), *Tcu*DyP (**B**), *Aau*DyP (**C**), VPL (**D**) and LiPH8 (**E**) and control without enzyme (**F**) in 100 mM tartrate, pH 3, containing 1 mM H_2O_2. The reactions, which were incubated for 1 h at 25 °C and 300 rpm (and stopped with sodium azide before analysis) revealed the appearance of a peak with RT 8.23-8.24 min (in **C**-**E**) identified as veratraldehyde (VAd) based on its mass and UV-vis spectra.

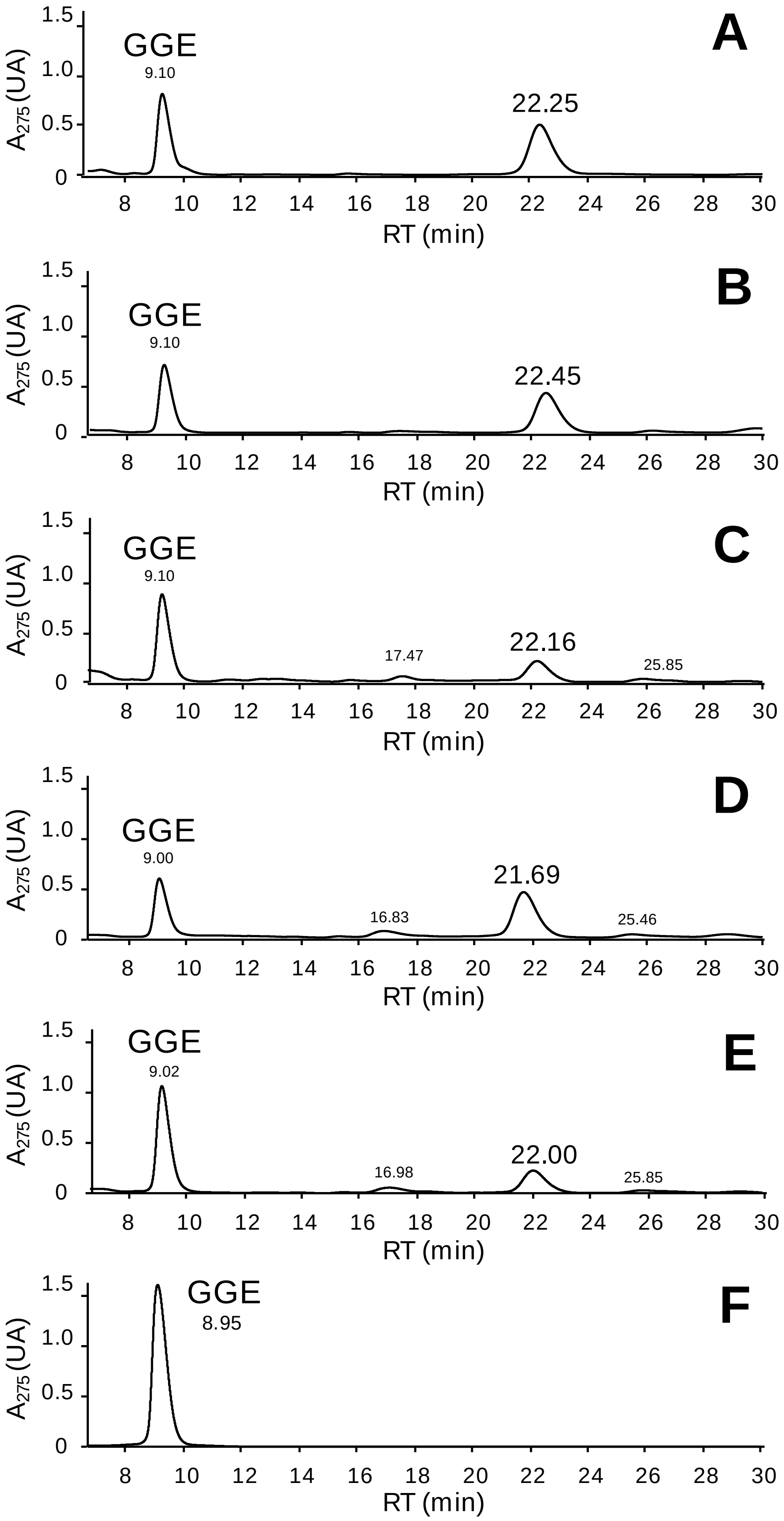

FIGURE S5. LC analysis (275-nm profiles) of GGE (1 mM) reactions with 40 μM *Asp*DyP2 (**A**), *Tcu*DyP (**B**), *Aau*DyP (**C**), VPL (**D**) and LiPH8 (**E**) and control without enzyme (**F**) in 100 mM tartrate, pH 3, containing 0.6 mM H_2O_2. The reactions, which were incubated for 1 h at 25 °C and 300 rpm (and stopped with sodium azide before analysis), revealed the decrease of the GGE peak and the appearance of a peak with RT 22.00-22.45 min, whose mass spectrum suggests a substrate dimerization product.

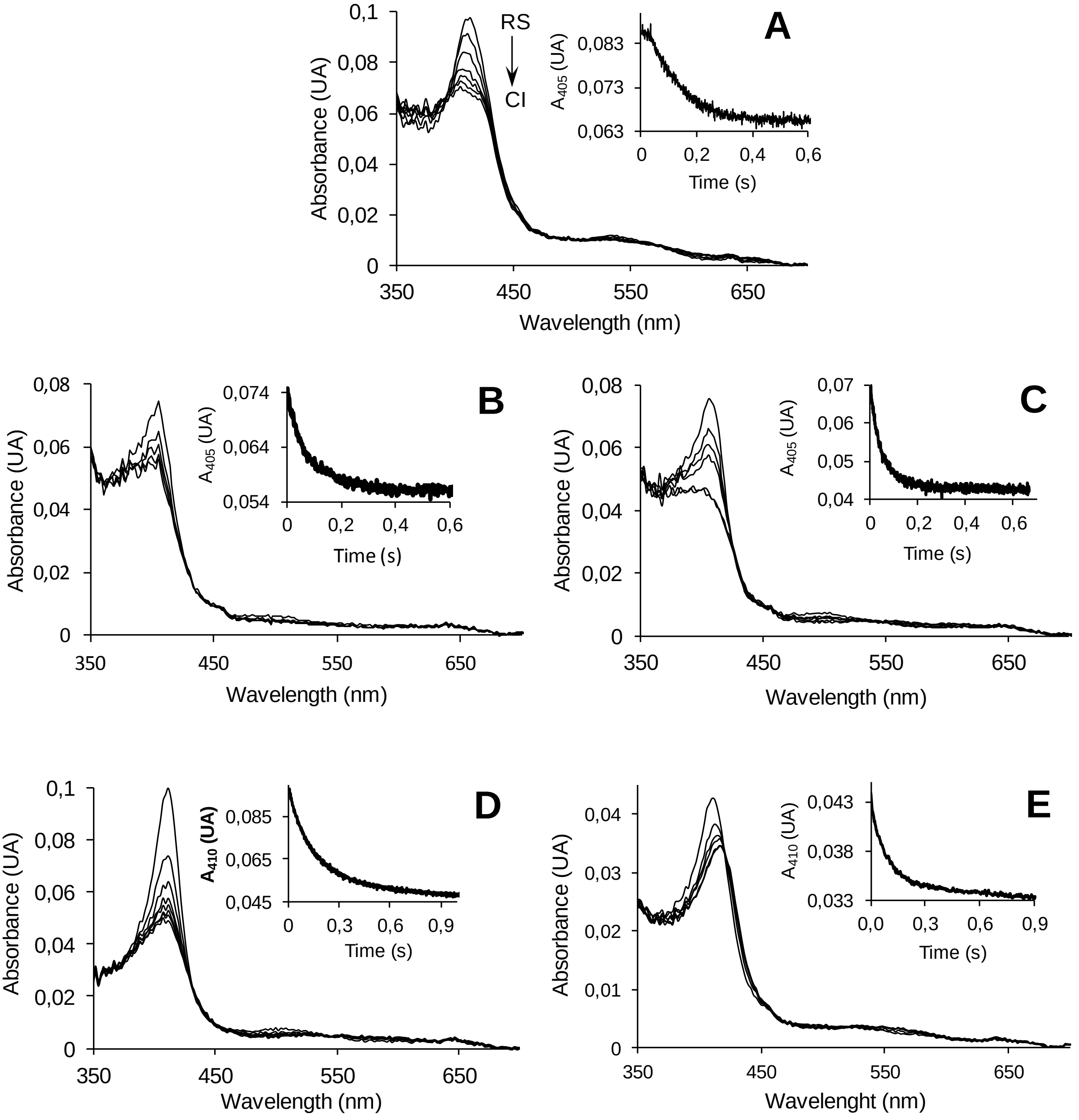

FIGURE S6. Spectral changes during CI formation by H_2O_2 addition to *Asp*DyP2 (**A**), *Tcu*DyP (**B**), *Aau*DyP (**C**), *Per*VPL (**D**) and *Pch*LiPA (**E**). The insets show time traces near the Soret maximum (at 405 nm for DyPs, and 410 nm for *Per*VPL and *Pch*LiPA) to attain equilibrium conditions. All reactions were at optimal pH 3, and 25 °C.

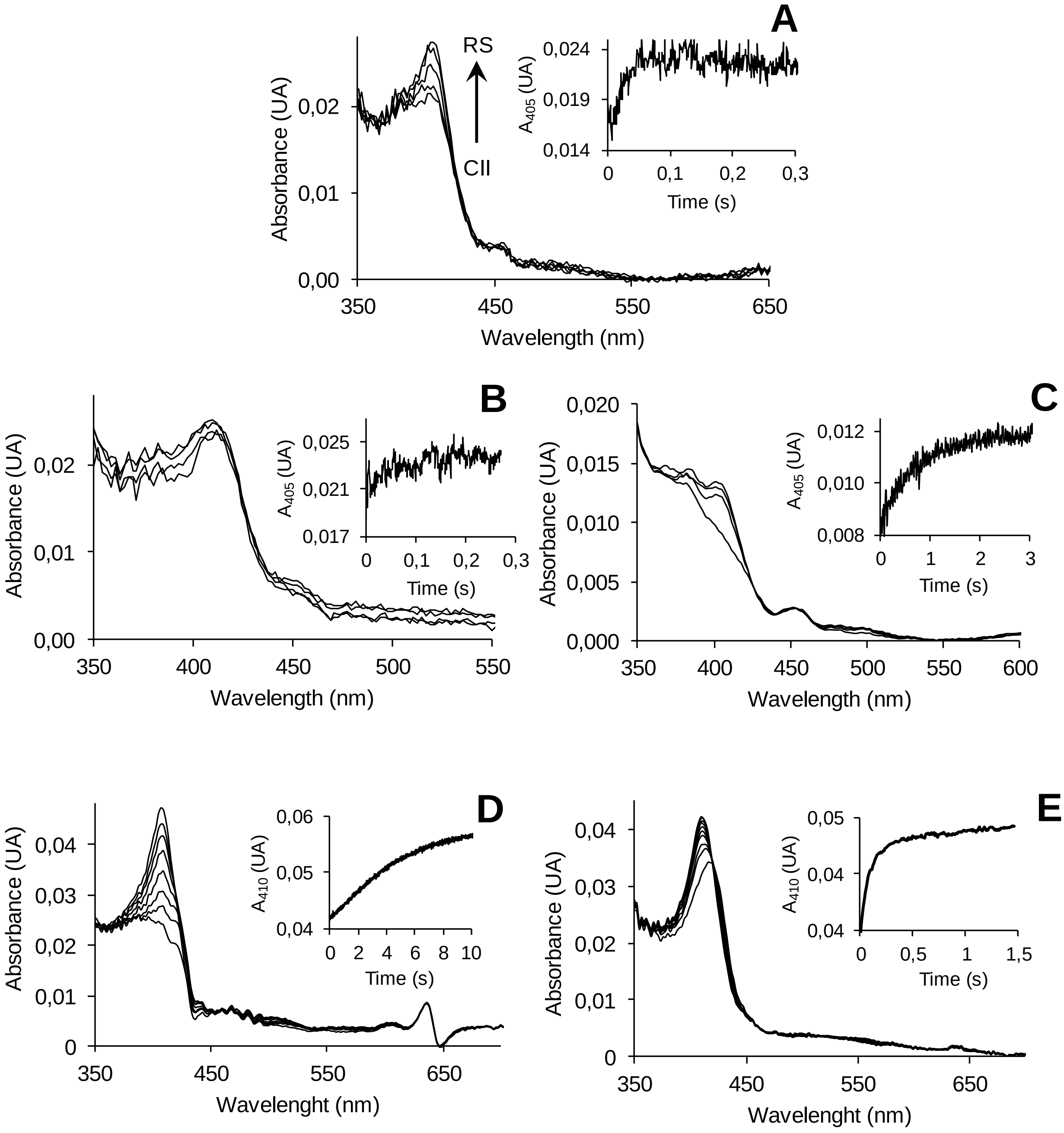

FIGURE S7. Spectral changes upon reduction with tyrosine of CII species formed by adding H_2O_2 and one equivalent of FeKCN to *Asp*DyP2 (**A**), *Tcu*DyP (**B**), *Aau*DyP (**C**), *Per*VPL (**D**) and *Pch*LiPA (**E**). The insets show time traces near the Soret maximum (at 405 nm for DyPs, and 410 nm for *Per*VPL and *Pch*LiPA) to attain equilibrium conditions. All reactions were at optimal pH 3, and 25 °C.
